# Multiomic analysis identifies natural intrapatient temporal variability and changes in response to systemic corticosteroid therapy in chronic rhinosinusitis

**DOI:** 10.1002/iid3.349

**Published:** 2020-11-21

**Authors:** Michael Hoggard, Bincy Jacob, David Wheeler, Melissa Zoing, Kevin Chang, Kristi Biswas, Martin Middleditch, Richard G. Douglas, Michael W. Taylor

**Affiliations:** ^1^ School of Biological Sciences The University of Auckland Auckland New Zealand; ^2^ Auckland Science Analytical Services The University of Auckland Auckland New Zealand; ^3^ Nextgen Bioinformatic Services Palmerston North New Zealand; ^4^ Department of Surgery, School of Medicine The University of Auckland Auckland New Zealand; ^5^ Department of Statistics, Statistical Consulting Centre The University of Auckland Auckland New Zealand; ^6^ Maurice Wilkins Centre for Molecular Biodiscovery The University of Auckland Auckland New Zealand; ^7^ Present address: Department of Primary Industry Orange NSW Australia

**Keywords:** chronic rhinosinusitis, corticosteroids, mucosa, multiomics, polyposis

## Abstract

**Introduction:**

The pathophysiology and temporal dynamics of affected tissues in chronic rhinosinusitis (CRS) remain poorly understood. Here, we present a multiomics‐based time‐series assessment of nasal polyp biopsies from three patients with CRS, assessing natural variability over time and local response to systemic corticosteroid therapy.

**Methods:**

Polyp tissue biopsies were collected at three time points over two consecutive weeks. Patients were prescribed prednisone (30 mg daily) for 1 week between Collections 2 and 3. Polyp transcriptome, proteome, and microbiota were assessed via RNAseq, SWATH mass spectrometry, and 16S ribosomal RNA and ITS2 amplicon sequencing. Baseline interpatient variability, natural intrapatient variability over time, and local response to systemic corticosteroids, were investigated.

**Results:**

Overall, the highly abundant transcripts and proteins were associated with pathways involved in inflammation, FAS, cadherin, integrin, Wnt, apoptosis, and cytoskeletal signaling, as well as coagulation and B‐ and T‐cell activation. Transcripts and proteins that naturally varied over time included those involved with inflammation‐ and epithelial–mesenchymal transition‐related pathways, and a number of common candidate target biomarkers of CRS. Ten transcripts responded significantly to corticosteroid therapy, including downregulation of *TNF, CCL20*, and *GSDMA*, and upregulation of *OVGP1*, and *PCDHGB1*. Members of the bacterial genus *Streptococcus* positively correlated with immunoglobulin proteins IGKC and IGHG1.

**Conclusions:**

Understanding natural dynamics of CRS‐associated tissues is essential to provide baseline context for all studies on putative biomarkers, mechanisms, and subtypes of CRS. These data further our understanding of the natural dynamics within nasal polypoid tissue, as well as local changes in response to systemic corticosteroid therapy.

## Graphical abstract

The pathophysiology and temporal dynamics of affected tissues in chronic rhinosinusitis (CRS) remain poorly understood. This study comprised a comprehensive multiomics analysis of polyp biopsies from three patients with chronic rhinosinusitis with nasal polyps, assessing natural variability over time as well as local response to systemic corticosteroids. Overall, these data provide further support for current hypotheses of CRS and polyposis pathogenesis, provide essential temporal context to studies on biomarkers and mechanisms of polyposis, and highlight areas of focus for future targeted therapeutic options.

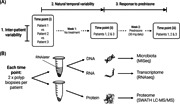

## 1 INTRODUCTION

Chronic rhinosinusitis (CRS) is a debilitating upper respiratory chronic inflammatory condition.^1^, ^2^ Leading hypotheses on the pathophysiology of CRS include deficiencies in local innate immunity and epithelial dysregulation.^3^, ^4^, ^5^, ^6^ Recent research has also implicated the sinonasal microbiota both in driving inflammation and influencing distinct inflammatory types of CRS.^7^, ^8^, ^9^, ^10^, ^11^ Nonetheless, the mechanisms underlying the etiology and ongoing pathophysiology of CRS remain poorly understood.

CRS is traditionally divided into two forms depending on the presence of nasal polyposis.^1^ More recently, CRS is increasingly thought of as a range of distinct conditions sharing similar clinical presentation, but with different underlying inflammatory mechanisms.^3^, ^9^, ^12^, ^13^, ^14^, ^15^, ^16^, ^17^, ^18^, ^19^, ^20^ Markers differentiating between different putative inflammatory endotypes of CRS have been identified, as well as multiple concurrent inflammatory types observed in some patients.^9^, ^14^, ^16^, ^19^, ^20^, ^21^, ^22^ Importantly, very little is known about the dynamics of processes within the mucosa or nasal polyp tissue over time. Better understanding the natural variation in CRS‐associated tissue will be vital for research aimed at clarifying the molecular mechanisms of CRS and nasal polyposis, and for informing treatment decisions.

Following the diagnosis of CRS, standard medical management includes saline irrigation, oral antibiotics, and topical and oral corticosteroids.^23^, ^24^, ^25^ While there is little evidence for the efficacy of topical antibiotics for most CRS patients,^26^ the benefits of local and systemic corticosteroid therapy are well supported, particularly in CRS with nasal polyps (CRSwNP) patients.^2^, ^6^, ^12^, ^26^, ^27^, ^28^, ^29^, ^30^ Local corticosteroids are particularly well tolerated, but limited accessibility to the inflamed sinonasal mucosa reduces the efficacy of topical treatments before surgical intervention.^31^ In comparison, potential deleterious side effects of long‐term systemic corticosteroid use limits their recommended application in CRS to short‐term management of CRSwNP.^2^, ^32^ It was of interest to examine the local mechanisms of action of systemic corticosteroids, with the aim of identifying targets for the development of novel therapies that achieve similar patient benefits without the need for, or sustained use of, systemic corticosteroids.

The aim of this study was to undertake a comprehensive multiomics assessment of nasal polyp tissue transcriptome, proteome, and associated bacterial and fungal microbiota, in three patients with CRSwNP. For all metrics, baseline interpatient variability, natural intrapatient variability over time (1 week), local response to systemic corticosteroid therapy (prednisone, 30 mg daily for 1 week), and interactions between the four data sets were investigated.

## 2 MATERIALS AND METHODS

### 2.1 Patient recruitment and sample collection

Three patients with CRSwNP listed for bilateral functional endoscopic sinus surgery for CRS were recruited. All patients were male, of New Zealand European ancestry, nonsmokers, aged 46–59 years, and with Lund–Mackay clinical severity scores ranging 17 to 23 (Table S1). None of the patients had taken antibiotics or corticosteroids in the 4 weeks before the study. This study was approved by the New Zealand Health and Disability Ethics committee (14/NTA/134), and written informed consent was obtained from all participants.

**Figure 1:**
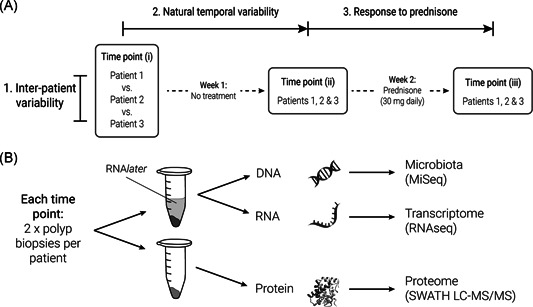
Experimental design and sampling. (A) Sample collection was designed to enable investigation of each of the following: (1) interpatient variability (patients 1 vs. 2 vs. 3); (2) natural temporal variability (time points i vs. ii); and (3) treatment effects of corticosteroids (prednisone). In total, two biopsies were collected from each patient at each respective time point, resulting in 18 biopsy samples. (B) At each time point, two nasal polyp biopsies were collected from each patient, with one biopsy placed into RNA*later* and one into an empty collection tube. DNA and RNA were extracted in parallel from biopsies collected in RNA*later* and prepared for microbiota (bacterial 16S ribosomal RNA and fungal ITS2; MiSeq) and transcriptome analyses (RNAseq), respectively. Remaining biopsies were prepared for proteome analysis (SWATH LC‐MS/MS)

Samples were collected at three time points over two consecutive weeks (time points i, ii, and iii; Figure 1A). Between time points ii and iii, patients were prescribed oral prednisone (30 mg daily) for 1 week. Sample collection was designed to enable investigation of each of the following: (1) baseline interpatient variability; (2) natural variability over time (time points i vs. ii); and (3) treatment effects of corticosteroids (prednisone; pretreatment time points vs. posttreatment [iii]). At each time point, two small adjacent nasal polyp tissue biopsies (<0.1 g, ~1 mm diameter) were collected. One biopsy, intended for human transcriptome and microbiota analyses, was placed into RNA*later* (Life Technologies). All samples were stored at −20°C until the time of sample processing.

### 2.2 Sample processing

#### 2.2.1 Transcriptome and microbiota community sequencing

DNA and RNA were extracted in parallel from polyp tissue biopsies collected in RNA*later* using the Qiagen AllPrep DNA/RNA Isolation Kit (Qiagen) as outlined in Supporting Information S1, Supplementary Methods, and as per manufacturer's instructions. DNase‐treated RNA for transcriptome analysis (RNAseq) was submitted to the sequencing provider (Auckland Genomics Ltd.) for final sample processing, library preparation, and sequencing on two lanes of an Illumina HiSeq machine (150 bp, paired‐end reads; Figure 1B). Extracted DNA was used for microbial community amplicon sequencing (Figure 1B). Bacterial 16S ribosomal RNA (rRNA) and fungal ITS2 genomic markers were polymerase chain reaction (PCR)‐amplified in triplicate using the primers 341F–785R,^33^ and ITS3–ITS4,^34^ as previously described,^35^, ^36^ and submitted to the sequencing provider (Auckland Genomics Ltd.) for library preparation and sequenced on the Illumina MiSeq platform (2 × 300 bp, paired‐end reads).

#### 2.2.2 Proteome (SWATH‐MS)

Remaining polyp biopsies (those not collected in RNA*later*) were processed and prepared for proteomic analysis as described in Supporting Information S1, Supplementary Methods. Liquid chromatography with tandem mass spectrometry (LC‐MS/MS) was conducted for each sample using SWATH acquisition, with fragment ion areas (and Benjamini and Hochberg false discovery rate [FDR] calculations for each ion) calculated by PeakView (v. 2.2) with the SWATH MicroApp 2.0 (Sciex; Figure 1B).

### 2.3 Data processing

#### 2.3.1 Transcriptome (RNAseq)

Raw RNAseq reads (>185 million/sample) were processed using BBDuk^37^ (v. 36.86) to remove sequencing adapters and low‐quality base calls (Phred score < 10). The high‐quality reads were then mapped to the Ensembl Human genome (v. GRCh38)^38^ using HISAT2 (v. 2.0.5),^39^ and read counts allocated to the Ensembl human gene models (GRCh38.94) by HT‐Seq (v. 0.6.0)^40^ using the “Union mode.”

#### 2.3.2 Proteome (SWATH‐MS)

Data for each patient (three samples each) were processed independently using Excel, as described in Supporting Information S1, Supplementary Methods. Sums of fragment areas for each peptide were calculated, followed by peptide area sums for each protein. Protein area sums were used for all subsequent analyses.

#### 2.3.3 Microbiota (16S rRNA and ITS2 marker amplicon sequencing)

Bioinformatics processing of 16S rRNA gene and ITS2 marker amplicon sequences was conducted in USEARCH (v. 10),^41^, ^42^, ^43^, ^44^ generating taxonomically assigned^45^, ^46^ zero‐radius operational taxonomic units (ZOTUs; based on 100% sequence similarity, and analogous to amplicon sequence variants), as previously described^36^, ^47^ (further described in Supporting Information S1, Supplementary Methods). Alpha diversity indices (richness, Shannon's diversity, and Simpson's evenness) were calculated in USEARCH.

### 2.4 Data analyses and statistics

An initial exploratory analysis of natural transcript and protein variability over time was conducted by calculating ratios of normalized raw read counts between the first and second time points (times i and ii) for each individual patient (excluding transcripts with read counts < 1000, as these can misrepresent the scale of fold differences).

#### 2.4.1 Transcriptome

The R package DESeq2 (v. 1.14.1)^48^, ^49^ was used to identify differentially expressed genes (DEGs) based on the read count data, as described in the package's vignette. The following RNAseq comparisons were used: (1) natural variability (time point i vs. ii); and (2) response to treatment (ii vs. iii). Reported significant DEGs are based on FDR‐adjusted *p* values (*α* = .05). Ensembl IDs were converted to HUGO Gene Nomenclature Committee (HGNC) symbols for ease of interpretation and to standardize reporting between RNAseq and proteome results.

#### 2.4.2 Proteome

Log‐transformed (natural log) data were tested for the following: (1) baseline interpatient differences; (2) natural variability; and (3) treatment effects (prednisone). Linear models were fitted for each protein, and tested using one‐way analysis of variance (ANOVA). For “natural variability” and “treatment” comparisons, linear mixed‐effects models were fitted with the addition of interpatient differences fitted as random effects. For “interpatient” (patient 1 vs. 2 vs. 3) and “natural variability” (times i vs. ii vs. iii), post‐hoc testing of variables with ANOVA *p* < .05 was conducted via two‐sample *t* tests and Tukey's honest significant difference tests (Tukey's *p* value, *α* = .05). For “treatment” (“control” vs. “treatment”), ANOVA results are reported (ANOVA *p*‐value, *α* = .05). UniProt IDs were converted to HGNC symbols to standardize reporting between proteome and RNAseq results.

#### 2.4.3 PANTHER analyses and pathways of interest data subset (“pathways_subset”)

Molecular pathways and Gene Ontology (GO) terms enriched for DEGs and proteins (DEG with FDR‐adjusted *p* < .1; proteins with Tukey's *p* < .05) for natural variability and response to prednisone were identified using PANTHER (v. 14.0)^50^, ^51^ via the functional classification tool and statistical overrepresentation test (release: 2018‐11‐13; FDR‐adjusted *p* value, *α* = .05), using GO database release 2018‐12‐01.^52^

To perform more focused subanalyses investigating biologically relevant mechanisms likely to be involved in CRS processes, a subset of genes (898 genes) were identified based on their involvement in a subset of selected PANTHER pathways of interest (herein referred to as “pathways_subset”; Table 1). Transcript and protein data subsets were established for 813 pathways_subset‐matching transcripts, and 66 pathways_subset‐matching proteins, respectively.

**Table 1:** PANTHER pathways of interest subset (pathways_subset)

	Associated genes^a^
Apoptosis signaling	118
B‐cell activation	43
Blood coagulation	34
Cadherin signaling	157
Inflammation mediated by chemokine and cytokine signaling	256
Interferon‐gamma signaling	30
Interleukin signaling	89
T‐cell activation	92
TGF‐beta signaling	99
Toll receptor signaling	57
Wnt signaling	311
**Unique genes associated with all subset pathways**	**898**
**Transcriptome pathways_subset**	**813**
**Proteome pathways_subset**	**66**

^a^ Number of genes associated with each of the PANTHER pathways of interest (filtered by species: *Homo sapiens*). Unique genes across all 11 pathways of interest are given in bold, together with the number of matches in the transcriptome and proteome data sets (comprising the final “transcriptome pathways_subset” and “proteome pathways_subset” data sets, respectively).

#### 2.4.4 Combined data

The remaining analyses were conducted in R (v. 3.3.0)^48^ using log‐transformed normalized read counts for each data set. The 20 most abundant bacterial ZOTUs and fungal ZOTUs, and microbiota diversity indices were compared between (1) patients and (2) time points (i, ii, and iii) via Kruskal–Wallis tests with FDR adjustment, with post‐hoc pairwise testing of significant variables via Dunn's test of multiple comparisons (*p* values) together with FDR adjustment (*α* = .05). Bray–Curtis dissimilarities were calculated for all data sets using the vegan package (v. 2.5‐1).^53^ Hierarchical clustering analyses and ordination analyses (nMDS, beta‐dispersion, and adonis) were conducted based on Bray–Curtis dissimilarities. Beta‐dispersion analyses were calculated separately for each grouping of “patient” and “treatment” (none were significantly differently dispersed [*p* > .05]). Adonis incorporated patient differences first, followed by treatment.

Spearman's correlation analyses were calculated for the following: (1) the 20 most abundant variables from each data set; and (2) variables shared between transcriptome and proteome data sets based on matching HGNC IDs (proteins with incomplete data sets across all nine samples were excluded). For the latter, correlation analyses were conducted for each matching data pair (comparing transcription data with its related protein), with a focus on negative correlation patterns. Correlation analyses included pairwise testing of significance via cor_pmat(), as well as FDR adjustment calculation (*α* = .05 in both cases).

Heat maps of log‐transformed data were generated with hierarchical clustering of the variables. Transcriptome and proteome variables were divided into several clusters (depending on the number of variables) to simplify the presentation of individual transcripts/proteins and associated PANTHER pathways. For visual clarity, figures are color‐coded throughout as follows: transcriptome data, red; proteome data, purple; bacterial data, green; fungal data, yellow.

### 2.5 Data availability

Transcriptome and microbiota raw sequence data have been uploaded to the SRA‐NCBI repository (BioProject accessions: PRJNA608823^54^ and PRJNA608821^55^).

Further details on materials and methods are provided in Supporting Information S1, Supplementary Methods.

## 3 RESULTS

### 3.1 General overview

In total, 58,734 distinct messenger RNA (mRNA) transcripts were identified (range, 18,107–24,634/sample). The genes with the highest normalized read counts in the transcriptome data set encoded members of the prostaglandin‐endoperoxide synthase family (*COX1, COX2, COX3*), and those involved in the electron transport chain (*ND1, ND2, ND4, ND5, CYTB, ATP6*; Figure S1). Proteomics data identified 4345 peptides, comprising 921 proteins. Proteins with the highest signal in the data (normalized counts) included the blood protein ALB, globin proteins (HBB and HBA1), cellular and tissue structural proteins (VIM, COL1A1, COL1A2, COL6A3, and HIST1H4A), and immunoglobulin proteins (IGKC and IGHG1; Figure S2). Additional highly abundant variables of interest included transcripts for the genes *ALOX15, POSTN, S100A11*, and *CST1*, and the proteins COL3A1, COL6A1, FGA, FGB, FGG, FN1, S100A8, S100A9, and S100A11.

**Figure 2:**
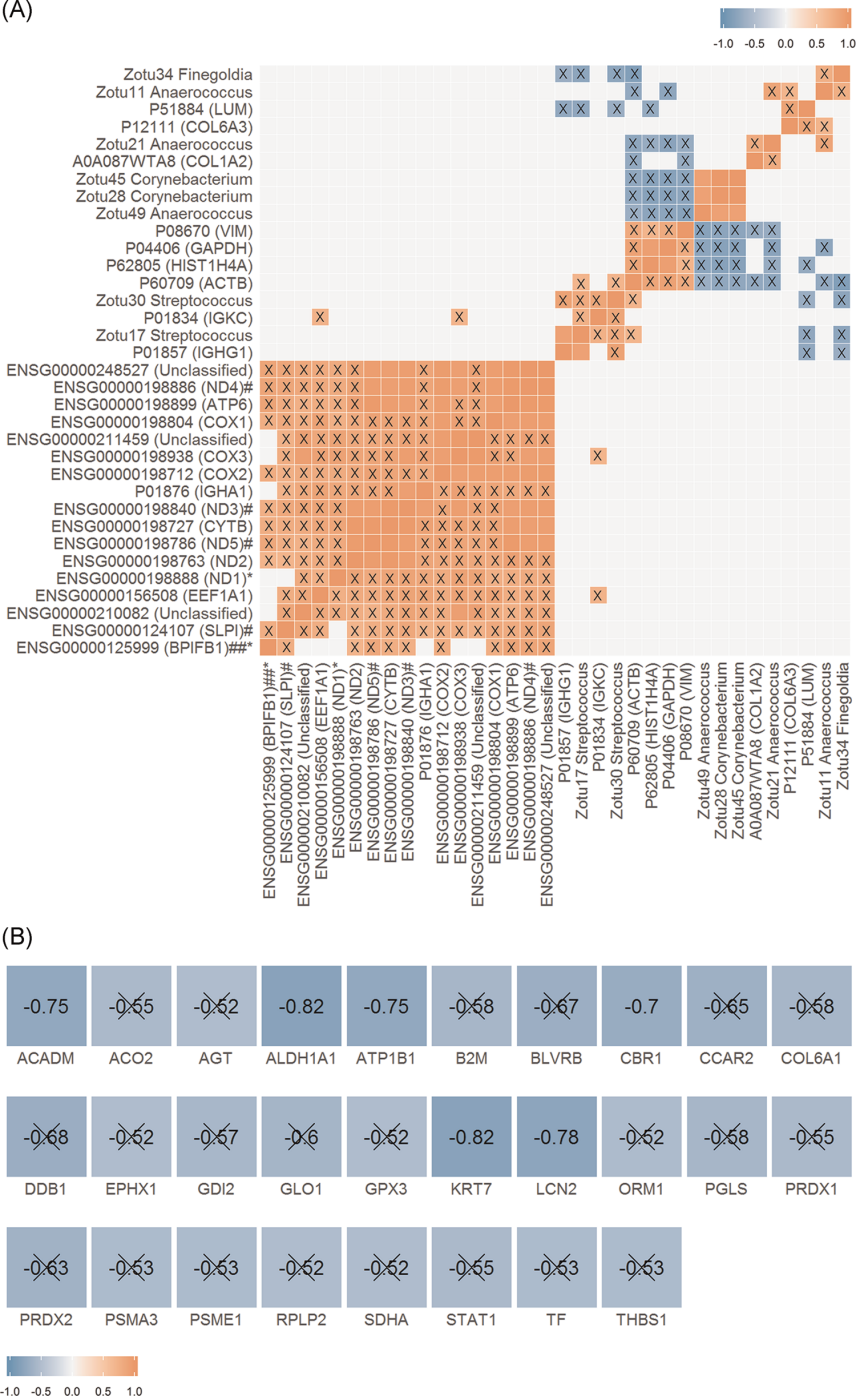
Spearman's correlation analyses. Heat maps of pairwise Spearman's correlation analyses with color coding reflecting Spearman's correlation coefficient (*ρ*; blue, −1; red, 1). (A) Correlation analysis including the 20 most abundant variables from each data set (transcriptome, proteome, bacterial 16S rRNA, and fungal ITS2). All (80) variables were included in the analysis and a subset of significant correlations is presented. Pairwise correlations were tested via the cor_pmat() function in R, and Benjamin–Hochberg false discovery rate (FDR) was calculated. Nonsignificant correlations (unadjusted *p* > .05) are uncolored. X overlay = correlations that were no longer significant following FDR adjustment (FDR‐adjusted *p* > .05). ^#^Variables with unadjusted *p* < .05 and ^##^FDR‐adjusted *p* < .05 in testing between time points i and ii (natural variability). *Unadjusted *p* < .05 and **FDR‐adjusted *p* < .05 in testing between time points ii and iii (response to corticosteroids). (B) Moderate to strong negative associations (−1 < Spearman's *ρ* < −0.5) between transcripts and the protein into which they translate (based on matching HGNC ID). Pairwise correlations were tested via the cor_pmat() function. X overlay = nonsignificant following pairwise significance testing (unadjusted *p* > .05)

Abundant bacterial genera included *Staphylococcus, Corynebacterium, Dolosigranulum, Anaerococcus*, and *Propionibacterium* (Figure S3A). The most abundant fungal genera included *Malassezia, Candida, Rhodotorula*, and unclassified members of Malasseziales, Dothideomycetes, Mycosphaerellaceae, and Phaeophaeriaceae (Figure S3B). In correlation analyses, a handful of microbial–protein associations were observed (Figure 2A). Bacterial ZOTUs of *Corynebacterium* and *Anaerococcus* were negatively associated with the proteins VIM, HIST1H4A, ACTB, and GADPH (−0.9 < *ρ* < −0.7). Two ZOTUs of *Anaerococcus* positively correlated with collagen proteins COL6A3 and COL1A2 (*ρ* = 0.8 and 0.7, respectively), and two ZOTUs of *Streptococcus* positively correlated with immunoglobulin proteins IGKC and IGHG1 (0.8 < *ρ*). Of these microbial associations, only that between *Streptococcus* and IGHG1 remained significant after FDR adjustment.

In total, 433 loci were shared between the transcriptome and proteome data sets. Six pairs of mRNAs and their proteins were significantly moderately or strongly negatively correlated (−1.0 < *ρ* < −0.5), including *ACADM, ALDH1A1, ATP1B1, CBR1, KRT7*, and *LCN2* (Figure 2B).

### 3.2 Interpatient differences

In pairwise comparisons, 279 proteins significantly differed between two or more patients at baseline (Collections i and ii). Notable differences included IGHG3, and the eosinophilia‐related proteins EPX, PRG2, and RNASE3 (up to 38–112 times difference between some patients). Patient 1 also had concomitant asthma, and a considerable proportion of the significant interpatient comparisons were in relation to Patient 1 (vs. Patient 2 or 3). Twenty‐six pathways_subset proteins (as per the pathways of interest listed in Table 1) were significantly different between patients in pairwise comparisons, including RNASE3, COL14A1, ALOX15, and the coagulation factor F13A1 (Table S2). Few interpatient significant differences were identified for bacterial and fungal community data.

In hierarchical clustering analysis, samples tended to cluster by patients for protein (including the pathways_subset) and bacterial community data (Figure S4), indicating stronger inter‐ than intrapatient differences over time. In adonis analyses, interpatient differences significantly explained 69% and 73% of the protein and pathways_subset protein data, respectively, and 43% of the bacterial data (*p* = .007, .006, and .014, respectively). Patient differences may also explain up to 30% of the transcriptome data variability; however, this was outside the significance threshold (*p* = .067).

### 3.3 Natural variability over time

In one or more patients, the expression of 191 transcripts and abundances of 24 proteins changed by a ratio of greater than 5:1 naturally over time (between time points i and ii; Tables 2 and S3). Markers of note included transcripts and proteins for *CST1* and *POSTN*; transcripts for *SPRR1A, SPRR1B, SPRR3, KRT6A, DSG3, TCN1, AQP1, MUC5B, MUC5AC, VIM, S100A2, S100A14*, and *LAMB1*; and proteins DEFA1, KRT14, FGA, FGB, FGG, S100A8, S100A9, EPX, CLC, IGHV1‐18, and PRG2. Several pathways_subset transcripts (as per the pathways of interest listed in Table 1) also changed notably (greater than 3:1) in one or more patients, including *CXCL8* (IL‐8), *A2M, CCL18, GDF15, TNFAIP3*, and *CD14* (Table 2).

**Table 2:** Natural intrapatient transcriptome and proteome variability over the course of 1 week^a^

	Ratio difference (time i vs. ii)		
	Patient 1	Patient 2	Patient 3
Transcriptome (pathways_subset)			
ENSG00000129538_(*RNASE1*)	3.0	−24.4	−4.3
ENSG00000169429_(*CXCL8*)	−3.3	13.4	−4.6
ENSG00000175899_(*A2M*)	1.3	−9.3	−6.3
ENSG00000142156_(*COL6A1*)	−1.4	−11.4	−1.9
ENSG00000175592_(*FOSL1*)	−7.5		2.0
ENSG00000275385_(*CCL18*)	−3.1	−7.3	−1.7
ENSG00000110799_(*VWF*)	1.4		−6.2
ENSG00000142173_(*COL6A2*)	−3.0	−3.3	−4.3
ENSG00000130513_(*GDF15*)	−2.0	5.4	1.9
ENSG00000184557_(*SOCS3*)	1.1		−5.1
ENSG00000181085_(*MAPK15*)	3.6	3.6	1.6
ENSG00000118503_(*TNFAIP3*)	−1.9	4.1	−2.4
ENSG00000170458_(*CD14*)	−1.7	−1.0	−4.9
ENSG00000162552_(*WNT4*)	3.2	1.9	2.1
ENSG00000122861_(*PLAU*)	−4.2	1.8	1.1
ENSG00000115415_(*STAT1*)	−2.2	3.5	1.3
ENSG00000102908_(*NFAT5*)	−1.3	−3.6	−1.8
ENSG00000181104_(*F2R*)	−1.0	−2.0	3.7
ENSG00000006210_(*CX3CL1*)	2.4	−3.1	1.1
ENSG00000271503_(*CCL5*)	−1.0	3.1	−2.2
ENSG00000083857_(*FAT1*)	1.1	−3.9	1.1
ENSG00000011422_(*PLAUR*)	−3.6	1.2	−1.1
Proteome			
P59665_(DEFA1)_(defensin alpha 1 (HGNC:2761))	9.5		
P49207_(RPL34)_(ribosomal protein L34 (HGNC:10340))	1.6		13.8
P02533_(KRT14)_(keratin 14 (HGNC:6416))	−6.8		
P62857_(RPS28)_(ribosomal protein S28 (HGNC:10418))	−5.6		
P02675_(FGB)_(fibrinogen beta chain (HGNC:3662))	−1.0	−13.9	−1.8
P05109_(S100A8)_(S100 calcium‐binding protein A8 (HGNC:10498))	1.0	−12.2	−2.2
P02671_(FGA)_(fibrinogen alpha chain (HGNC:3661))	−1.4	−9.6	−1.8
P02679_(FGG)_(fibrinogen gamma chain (HGNC:3694))	−1.6	−9.1	−2.0
B0YIW2_(APOC3)_(apolipoprotein C3 (HGNC:610))	−1.2	−6.3	−4.9
P00915_(CA1)_(carbonic anhydrase 1 (HGNC:1368))	−8.7	2.1	1.4
P11678_(EPX)_(eosinophil peroxidase (HGNC:3423))	−1.0	−1.1	9.2
P06702_(S100A9)_(S100 calcium‐binding protein A9 (HGNC:10499))	1.2	−7.8	−2.2
P02042_(HBD)_(hemoglobin subunit delta (HGNC:4829))	−7.4	1.7	1.1
Q05315_(CLC)_(Charcot–Leyden crystal galectin (HGNC:2014))	1.1	2.2	6.7
P01037_(CST1)_(cystatin SN (HGNC:2473))	−1.2	5.4	
P30838_(ALDH3A1)_(aldehyde dehydrogenase 3 family member A1 (HGNC:405))	−2.4	6.2	1.1
P69905_(HBA1)_(hemoglobin subunit alpha 1 (HGNC:4823))	−6.5	1.8	1.1
Q15063_(POSTN)_(periostin (HGNC:16953))	1.0	7.3	1.1
D6RGG3_(COL12A1)_(collagen type XII alpha 1 chain (HGNC:2188))	−1.5	5.7	1.9
Q8IUX7_(AEBP1)_(AE binding protein 1 (HGNC:303))	−1.2	5.4	2.4
P68871_(HBB)_(hemoglobin subunit beta (HGNC:4827))	−5.9	1.5	1.1
P08311_(CTSG)_(cathepsin G (HGNC:2532))	−1.0	−5.3	−1.8
A0A0C4DH31_(IGHV1‐18)_(immunoglobulin heavy variable 1‐18 (HGNC:5549))	1.1	−5.4	1.0
P13727_(PRG2)_(proteoglycan 2, pro eosinophil major basic protein (HGNC:9362))	1.2	−1.1	5.0

^a^ Ratios compare the difference between time points i and ii for each patient individually. Transcriptome variables from the pathways_subset data set with greater than three times difference within one or more patients, and proteins with greater than five times difference, are presented. Missing data reflect transcript comparisons where at least one of the normalized transcript counts was <1000 (which were excluded from this analysis), or where proteins were not detected in all three time points for that respective patient (and were excluded from the data). All transcripts with greater than five times difference in one or more patients are presented in Table S4.

**Figure 3:**
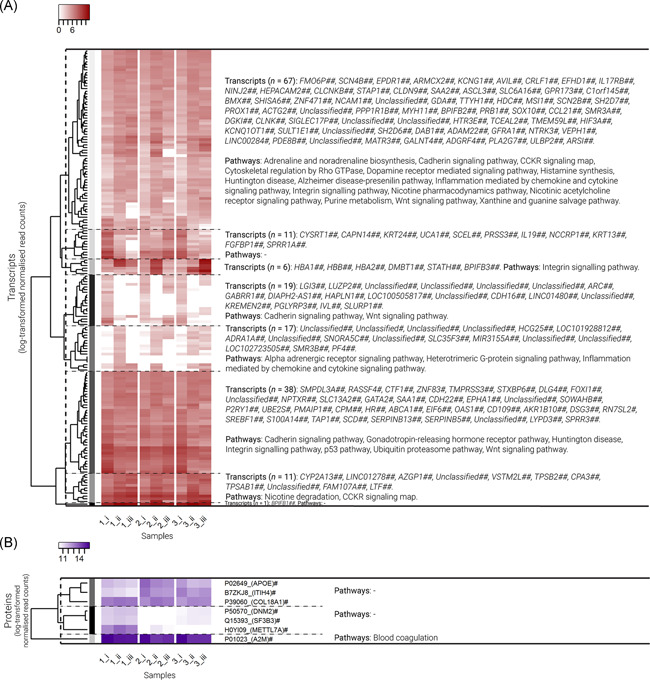
Transcriptome (differentially expressed gene [DEG]) and proteome natural temporal variability (time points i vs. ii). Heat maps of log‐transformed normalized read counts for (A) transcripts (DEG; presented in red), and (B) proteins (presented in blue) that varied naturally over time (pairwise testing between time points i vs. ii; *p* < .05). Variables are ordered via hierarchical clustering based on Bray–Curtis dissimilarity. Variables are divided into clusters (represented by the grayscale color coding at branch tips), and pathways associated with the variables in each cluster are presented on the right. Samples represent three patients (1, 2, and 3) at three time points over two consecutive weeks (i, ii, and iii). ^#^Unadjusted *p* < .05 and ^##^false discovery rate (FDR)‐adjusted *p* < .05 in testing between time points i and ii (natural variability). *Unadjusted *p* < .05 and **FDR‐adjusted *p* < .05 in testing between time points ii and iii (response to corticosteroids)

Testing across all patients, 162 DEGs and 7 proteins differed significantly between time points i and ii, including the genes *IL17RB, BMX, HIF3A, CCL21, LTF, IL‐19, S100A14, CLDN9, SAA1, SAA2*, and *CLCNKB*, and proteins APOE, ITIH4, COL18A1, DNM2, SF3B3, METTL7A, and A2M (Figure 3 and Table S4). Significant DEG between times i and ii from the pathways_subset data included *CCL21, ACTG2, KREMEN2, MYH11, CDH22, PF4*, and *CDH16* (Figure S5A).

In PANTHER overrepresentation testing based on DEG and proteins (each tested separately) that varied significantly between times i and ii, significantly enriched GO terms included a number of processes likely involved in inflammation or epithelial–mesenchymal transition (EMT), such as antimicrobial humoral response, extracellular matrix organization, extracellular exosome, cell adhesion, and cornification (Table 3).

**Table 3:** PANTHER overrepresentation testing based on transcripts (DEG) and proteins that significantly varied naturally over the course of 1 week (times i vs. ii)^a^

	Fold enrichment	FDR‐adjusted *p* value
Transcriptome		
GO biological process complete		
Cornification (GO:0070268)	11.2	.001
Antimicrobial humoral response (GO:0019730)	9.1	.019
Cell adhesion (GO:0007155)	3.3	.003
GO cellular component complete		
Endocytic vesicle lumen (GO:0071682)	27.9	.005
Plasma lipoprotein particle (GO:0034358)	12.9	.034
Cornified envelope (GO:0001533)	11.6	.005
Postsynaptic density membrane (GO:0098839)	7.9	.046
Ion channel complex (GO:0034702)	4.5	.008
Collagen‐containing extracellular matrix (GO:0062023)	4.1	.009
Glutamatergic synapse (GO:0098978)	3.8	.025
Cell surface (GO:0009986)	3.2	<.001
Extracellular exosome (GO:0070062)	2.2	.001
Intrinsic component of plasma membrane (GO:0031226)	2.0	.033
Proteome		
PANTHER pathways		
Integrin signaling pathway (P00034)	30.1	.020
GO molecular function complete		
Protein‐containing complex binding (GO:0044877)	10.5	.034
GO biological process complete		
Substrate adhesion‐dependent cell spreading (GO:0034446)	>100	.007
Extracellular matrix organization (GO:0030198)	29.1	.003
GO cellular component complete		
Laminin‐11 complex (GO:0043260)	>100	.002
Laminin‐10 complex (GO:0043259)	>100	.001
Synaptic cleft (GO:0043083)	>100	.006
Blood microparticle (GO:0072562)	40.0	.009
Extracellular exosome (GO:0070062)	6.4	.007
Endomembrane system (GO:0012505)	3.4	.047

Abbreviations: DEG, differentially expressed gene; FDR, false discovery rate; GO, Gene Ontology.

^a^ Variables with FDR‐adjusted *p* < .1 from time i versus ii testing were entered into PANTHER overrepresentation testing. Significantly enriched GO terms (FDR‐adjusted *p* < .05) are presented. Where multiple significant GO terms were hierarchically nested, the lowest rank GO term is presented here.

Subtle differences were observed in bacterial and fungal communities within each patient over time (times i vs. ii and ii vs. iii; Figures S3 and S6). However, in pairwise comparisons testing across all patients, there were no significant temporal differences for the 20 most abundant ZOTUs or diversity indices for bacterial or fungal community data.

### 3.4 Change in response to prednisone treatment

**Figure 4:**
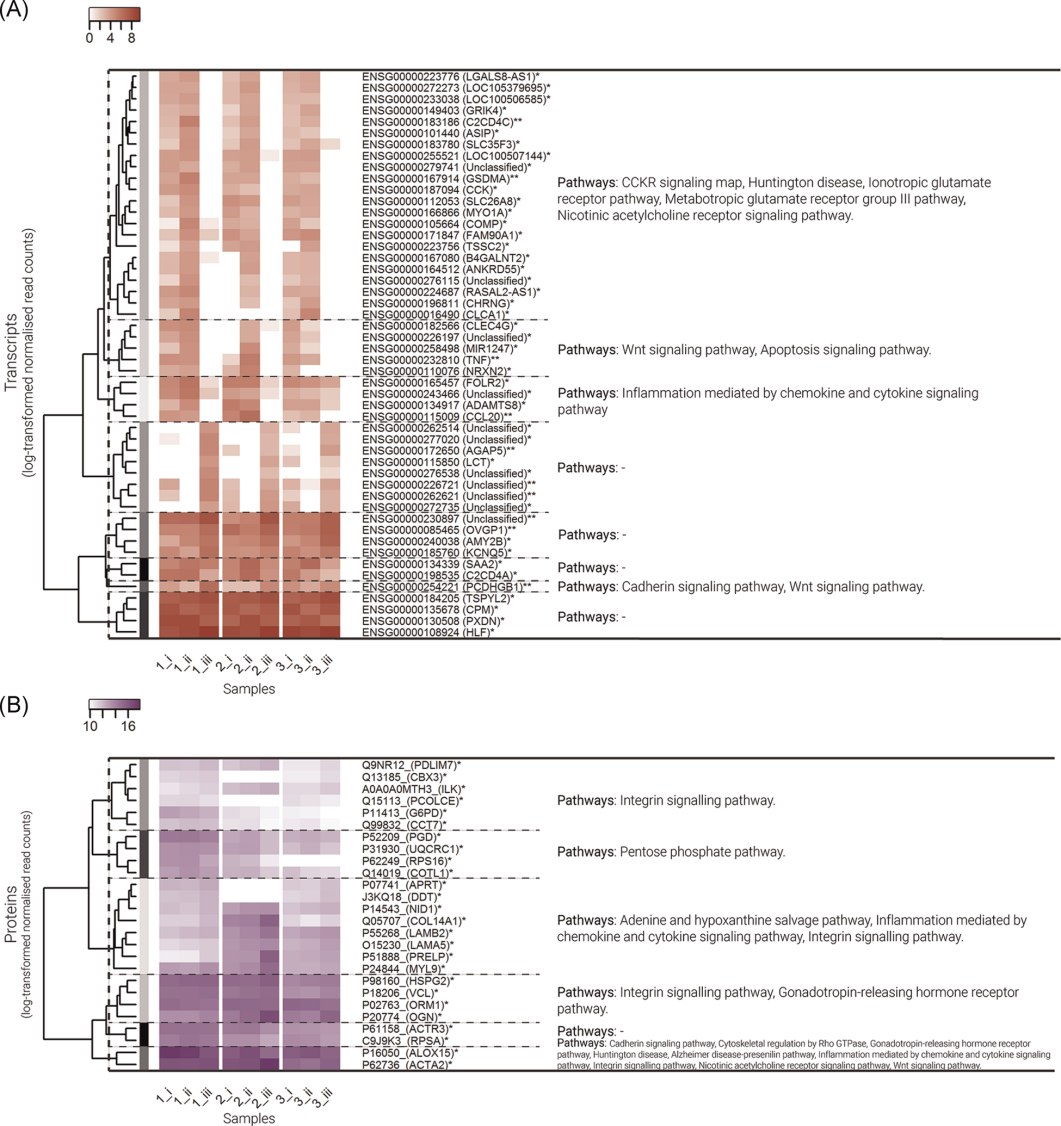
Transcriptome (differentially expressed gene [DEG]) and proteome response to systemic corticosteroid (prednisone) therapy (time points ii vs. iii). Heat maps of log‐transformed normalized read counts for (A) transcripts (DEG; presented in red), and (B) proteins (presented in blue) that varied in response to corticosteroid therapy (pairwise testing between time points ii versus iii; *p* < .05). Variables are ordered via hierarchical clustering based on Bray–Curtis dissimilarity. Variables are divided into clusters (represented by the grayscale color coding at branch tips), and pathways associated with the variables in each cluster are presented on the right. Samples represent three patients (1, 2, and 3) at three time points over two consecutive weeks (i, ii, and iii). ^#^Unadjusted *p* < .05 and ^##^false discovery rate (FDR)‐adjusted *p* < .05 in testing between time points i and ii (natural variability). *Unadjusted *p* < .05 and **FDR‐adjusted *p* < .05 in testing between time points ii and iii (response to corticosteroids)

Transcripts for 724 genes and 26 proteins differed between pre‐ and post‐corticosteroid (prednisone) treatment (times ii and iii; unadjusted *p* < .05). The 50 DEGs with the lowest *p* values are presented in Figure 4A. Protein changes included increases in LAMB2 (2:1), LAMB5 (1.6:1), OGN (3.3:1), COL14A1 (2.4:1), ACTA2 (2.1:1), and PRELP (3.5:1), and a decrease in ALOX15 (2.7:1) (unadjusted *p* < .05; Figure 4B). Of these, three were also among the pathways_subset proteins (ALOX15, ACTA2, and COL14A1; as per the pathways of interest subset listed in Table 1).

After FDR adjustment, 10 DEGs (but no proteins) were significant in response to prednisone treatment, including downregulation of *CCL20* (log_2_ fold change ratio = 10:1), *TNF* (9:1), *C2CD4C* (9:1), and *GSDMA* (9:1), and upregulation of *OVGP1* (2:1) and *PCDHGB1* (4:1; Figure 4A). Of these, three were also among the pathways_subset transcripts (*CCL20, TNF*, and *PCDHGB1*; Figure S5C).

In PANTHER overrepresentation testing based on DEG with FDR‐adjusted *p* < .1, no significantly enriched GO terms were identified after correction for multiple testing. Based on DEG with unadjusted *p* < .05 from DESeq2 analysis (724 transcripts), significantly enriched GO terms included predominantly inflammatory mechanisms. Significantly enriched GO terms associated with proteins that differed in response to corticosteroids included predominantly tissue and cellular structural mechanisms, including laminin‐11 complex, costamere, and cell–matrix adhesion (Table 4).

**Table 4:** PANTHER overrepresentation testing based on transcripts (DEG) and proteins that differed significantly in response to corticosteroid therapy (times ii vs. iii)^a^

	Fold enrichment	FDR‐adjusted *p* value
Proteome		
PANTHER pathways		
Integrin signaling pathway (P00034)	29.8	<.001
GO molecular function complete		
Extracellular matrix structural constituent conferring compression resistance (GO:0030021)	>100	.004
Protein‐containing complex binding (GO:0044877)	7.4	.001
GO biological process complete		
Pentose biosynthetic process (GO:0019322)	>100	.050
Platelet aggregation (GO:0070527)	55.1	.046
Nephron development (GO:0072006)	33.7	.006
Cell–matrix adhesion (GO:0007160)	25.8	.048
GO cellular component complete		
Laminin‐11 complex (GO:0043260)	>100	.002
Synaptic cleft (GO:0043083)	>100	.015
Costamere (GO:0043034)	85.0	.020
Stress fiber (GO:0001725)	38.5	.007
Lysosomal lumen (GO:0043202)	25.8	.017
Golgi lumen (GO:0005796)	24.2	.019
Focal adhesion (GO:0005925)	12.0	.002
Secretory granule lumen (GO:0034774)	10.1	.037
Extracellular exosome (GO:0070062)	7.3	<.001

Abbreviations: DEG, differentially expressed gene; FDR, false discovery rate; GO, Gene Ontology.

^a^ Variables with FDR‐adjusted *p* < .1 from time ii versus iii testing were entered into PANTHER overrepresentation testing. Significantly enriched GO terms (FDR‐adjusted *p* < .05) are presented. Where multiple significant GO terms were hierarchically nested, the lowest rank GO term is presented here.

Taken together, all data showed signs of change post corticosteroid (prednisone) treatment. However, the specific corticosteroid responses of individual patients varied considerably, and there was no obvious posttreatment profile “type” shared by individuals in the experiment (Figures S1–S3 and S6). This was further reflected in hierarchical clustering, where treatment samples did not cluster together (Figure S4), and adonis analyses, where treatment did not significantly explain any of the variability in the data.

### 3.5 PANTHER pathways

The most abundant transcripts and proteins included those associated with T‐cell activation, inflammation mediated by chemokine and cytokine, cadherin, integrin, and cytoskeletal signaling pathways, as well as Wnt, FAS, and apoptosis signaling (transcripts), and B‐cell activation and blood coagulation (proteins; Figures S1 and S2). Notably, DEG and proteins that varied naturally over time, and also those that differed in response to treatment, both included those associated with inflammation mediated by chemokine and cytokine, cadherin, integrin, and Wnt signaling pathways (Figures 3 and 4).

## 4 DISCUSSION

Establishing degrees of natural variability over time is essential to provide context for investigations of putative mechanisms and biomarkers of disease, and to inform treatment decisions. This study comprised a comprehensive multiomics analysis of nasal polyp biopsies from three patients with CRSwNP, assessing natural variability over time as well as local response to systemic corticosteroids.

### 4.1 The transcriptome and proteome of nasal polyp tissue in CRS

Abundant transcripts and proteins identified in this study included several involved in inflammation mediated by chemokine and cytokine, FAS, cadherin, integrin, Wnt, apoptosis, and cytoskeletal signaling, coagulation, and B‐ and T‐cell activation pathways. Processes associated with inflammation and tissue structural changes have similarly been highlighted previously as central mechanisms underlying CRS and/or nasal polyposis in CRSwNP, supporting the validity of the transcriptome and proteome data presented here.

EMT is associated with CRS.^6^ EMT includes increased production of matrix proteins such as collagens I, III, and IV, as well as fibronectin, laminin, vimentin, and periostin, and reduction in epithelial markers such as E‐cadherin.^6^, ^56^ Transcripts and/or proteins for vimentin (*VIM*), collagens *COL1A1, COL1A2, CO3A1, COL6A1, COL6A2*, and *COL6A3*, fibronectin (*FN1*), and periostin (*POSTN*) were all abundant in this study. Proteins involved in the coagulation cascade (including fibrinogen and fibronectin) have also been implicated in CRSwNP, with fibrin deposition (coupled with reduced fibrinolysis) implicated in the pathogenesis of nasal polyposis.^57^ Coagulation proteins FGA, FGB, FGG, and fibronectin (FN1) were all also abundant in nasal polyp biopsies in this study. These data lend further support to the hypotheses that aberrant EMT processes and coagulation cascade mechanisms play central roles in CRSwNP.^6^, ^57^

It was also of interest to investigate transcription and protein patterns in parallel. However, the resolution of each method differs considerably. In this study, over 58,000 transcripts were identified compared with 921 proteins. Low‐abundance proteins, including inflammatory cytokine signaling molecules, are generally omitted from the latter. Nonetheless, patterns were broadly consistent between the overlapping data sets. Exceptions included significant moderate to strong negative correlations between transcripts and their respective protein for ATPase Na^+^/K^+^ transporting subunit (*ATP1B1*), carbonyl reductase (*CBR1*), keratin (KRT7), and lipocalin *LCN2* (and a similar, but nonsignificant, pattern for *STAT1* and *COL6A1*). Posttranscriptional regulation can play important roles in disease, including some variants of cystic fibrosis^58^ (which often includes a CRS component), and maybe a further distinguishing factor of some variants of CRS.

### 4.2 Microbiota associations

Associations have previously been identified between the microbiota and inflammatory signaling and subtypes in CRS.^8^, ^9^, ^59^, ^60^, ^61^ In this study, few associations were observed between microbial data and nasal polyp transcription or proteins. High inter‐ and intrapatient microbiota variability is well described in CRS,^35^, ^62^, ^63^ and this may obscure some associations.

One association of note was a positive correlation between ZOTUs of *Streptococcus* and two immunoglobulin proteins (IGKC and IGHG1). *Streptococcus* spp. can produce IgG‐cleaving proteases,^64^, ^65^ and binding of *Streptococcus* M‐proteins to fibrinogen (abundant in nasal polyp tissue in this study) enables further IgG‐related immune evasion.^66^ *Streptococcus* spp. are known to be commonly associated with the sinonasal tract,^63^ however, their specific role in CRS remains unclear. *Streptococcus*‐mediated IgG inflammatory processes coupled with immune evasion may promote ongoing inflammation, while also providing strong selective pressure on the structure of the associated microbiota. Further study of possible roles of *Streptococcus* spp. in the pathogenesis of nasal polyposis is warranted.

### 4.3 Interpatient differences

In a stratified cohort of patients (all CRSwNP, adult male, of New Zealand European ancestry, and nonsmokers), numerous interpatient differences were observed, including prominent inflammation‐related proteins such as the immunoglobulin protein IGHG3, and the eosinophilia‐related proteins eosinophil peroxidase (EPX), proteoglycan 2 (PRG2; major basic protein), and eosinophilic cationic protein (RNASE3).

While corticosteroid therapy offers relief to a great number of CRSwNP patients, between 20% and 50% of patients are resistant.^12^, ^27^, ^67^, ^68^ Mucin 1 (*MUC1*) downregulation, elevated MUC4, and increased neutrophilia are markers of reduced response to corticosteroids.^67^, ^69^, ^70^ In contrast, eosinophilia is an important marker of improved responsiveness.^12^ Several markers of eosinophilia were among those that differed significantly between patients in this study, and recent studies provide support for distinguishing eosinophilic from noneosinophilic CRSwNP patients.^21^, ^22^ Concomitant asthma and/or eosinophilia may be key explanatory factors of interpatient and temporal intrapatient differences observed here (including response to treatment), and highlights the importance of the current focus on improved endotyping of CRS.^3^, ^9^, ^12^, ^13^, ^14^, ^15^, ^16^, ^17^, ^18^, ^20^

### 4.4 Natural variability over time

Identifying biomarkers and better delineating endotypes of CRS is a major focus of current research, and promises improved guidance of treatment decisions.^3^, ^9^, ^12^, ^13^, ^15^, ^16^ Several putative biomarkers have been identified.^9^, ^16^, ^57^, ^60^, ^71^, ^72^, ^73^, ^74^ However, very little is known about natural variability over time in CRS‐associated tissues. To our knowledge, this is the first study that has assessed changes occurring naturally over time in nasal polyp (or mucosal) tissue associated with CRS.

Between the first two time points (before the treatment phase) there were 162 significant DEGs, suggesting that many processes involved in CRS may vary substantially over relatively short time scales in the natural course of the disease. Numerous targets that may be considered promising candidates for CRS and/or nasal polyposis biomarkers (due to their potential roles in inflammation and tissue remodeling) showed natural variation within patients, including mucins (*MUC5B* and *MUC5AC*), cystatin SN (*CST1*), the S100 calcium‐binding *S100A8, S100A9*, and *S100A14*, alpha defensin (*DEFA1*), the eosinophilia‐related eosinophil peroxidase (*EPX*), proteoglycan 2 (*PRG2*; major basic protein), and Charcot–Leyden crystal galectin (*CLC*), coagulation‐related *FGA, FGB*, and *FGG*, claudin 9 (*CLDN9*), desmoglein (*DSG3*), periostin (*POSTN*), immunoglobulin component *IGHV1*, interleukin (IL)‐8 (*CXCL8*), *IL19, IL17RB*, chemokine ligands 18 and 21 (*CCL18* and *CCL21*), serum amyloids (*SAA1* and *SAA2*), keratin (*KRT6A* and *KRT14*), and vimentin (*VIM*). A number of these markers have been highlighted in previous cross‐sectional studies as putative biomarkers of CRS or subtypes of CRS.^9^, ^60^, ^71^, ^75^, ^76^, ^77^, ^78^, ^79^, ^80^

Increased variability in some markers may in itself be an important factor in CRS, as has been observed already for bacterial associations with CRS.^7^, ^10^, ^35^, ^60^, ^81^ It remains unclear whether the array of CRS subtypes currently hinted at^9^, ^13^, ^16^ are in fact distinct types of the condition, or simply phases of a continuum of varied parallel and dynamic inflammatory processes.^12^ Understanding the natural dynamics of CRS mucosa and nasal polyp tissue is essential to provide baseline context for all studies on biomarkers, mechanisms, and subtypes of CRS. To this end, recent studies identifying noninvasive proxies for tissue processes^75^, ^82^ are especially vital, and will enable large comprehensive time‐series investigation of sinonasal tissue processes in both CRS patients and healthy controls.

### 4.5 Change in response to prednisone treatment

Oral corticosteroids are recommended for the short‐term management of CRSwNP, but only as an infrequent recourse.^2^ Surprisingly, little is known of the local effects of systemic corticosteroid treatment on mucosal or nasal polyp tissue in CRS. The widespread effects observed here may be central to the efficacy of corticosteroids in diseases as immunologically complex as CRS. Nonetheless, this study has identified some markers and mechanisms that warrant investigation as candidates for targeted therapeutics.

Significant DEG due to treatment effects included marked downregulation of inflammatory mediators tumor necrosis factor (*TNF*), chemokine ligand 20 (*CCL20*), and gasdermin A (*GSDMA*; which is potentially involved in pyroptosis), and upregulation of markers that may reflect a reversal of processes of epithelial dysregulation, including the epithelial‐glycoprotein *OVGP1*, and cell adhesion molecule protocadherin gamma (*PCDHGB1*) transcription. Multiple changes in both pro‐inflammatory and anti‐inflammatory transcription have similarly been observed previously.^83^ These findings further support the hypotheses that aberrant inflammatory processes, epithelial dysregulation (including EMT), and/or coagulation cascade pathways,^5^, ^6^, ^57^ are key mechanisms in the pathogenesis of nasal polyposis. Therapies that target these specific effects locally, such as TNF inhibitors coupled with promoters of epithelial repair and differentiation, warrant further investigation.

### 4.6 Limitations

This study aimed to investigate nasal polyp‐associated mechanisms over time, including natural variation and change in response to corticosteroids (prednisone), in a comprehensive multilayered approach. Nasal polyp tissue was relatively accessible, enabling biopsy collection in the routine clinic setting over consecutive weeks. Nonetheless, patient recruitment for such a study is challenging, and ‐omics technologies remain relatively cost‐prohibitive for large cohort studies. As a result, this study is limited to three patients and is exploratory in nature. Inferences drawn from comparisons over large numbers of variables in a small patient group should be interpreted with caution. Furthermore, the study design was developed to enable repeated minimally invasive sampling in the clinic setting. Nonetheless, biopsy sampling is inherently invasive, and the local effects of wounding and wound healing over the course of each week may influence the degree of variability in tissue processes observed here. Results should not be interpreted as definitive markers of CRS (or response to treatment), but serve as an important proof of principle, and provide initial insights into markers worthy of more attention.

Each data set was generated from a single sample per patient per time point (one biopsy for proteomic analysis, and one biopsy for transcriptomic and microbiota analyses). The focus of this study was temporal intrapatient variability. However, comparably little is known about the natural spatial variability of processes within nasal polyp or mucosal tissue in CRS. The collection of additional biopsy samples at each time point was not feasible in this study, and it remains unclear whether the observed variability of some markers may be accounted for by spatial heterogeneity rather than temporal dynamics. The natural spatial variability of tissue processes represents an additional significant knowledge gap in the understanding of CRS and requires further study.

Considerable interpatient variability was observed in all four data sets, and generally patient differences more strongly partitioned the data than changes over time (including response to corticosteroid therapy). Interpatient differences in baseline local inflammatory mechanisms, and an individual response to corticosteroids, may have obscured some genuine associations. The effects of these differences will be especially pronounced due to the small sample size (three patients sampled over three time points). This study provides a template and highlights focal points for subsequent study in larger patient groups. In future, larger cohort temporal studies incorporating finer scale resolution of CRS subtypes (such as inflammatory endotypes) are required to further resolve genuine patterns of change over time.

Finally, the focus of this study was mechanisms specific to nasal polyposis within CRSwNP. Findings may not represent nonpolyp sinonasal mucosa processes in CRSwNP or chronic rhinosinusitis without nasal polyps (CRSsNP), and other mucosal markers may better differentiate between different variants of CRS.

Despite these limitations, however, this study contributes to several important findings. The observed interpatient variability and intrapatient dynamics both have implications for the interpretation of studies on biomarkers and mechanisms of CRS. Additionally, despite this background variability, several specific local effects of systemic corticosteroids were observed. These included transcripts and proteins related to several pathways previously identified as important in nasal polyp and CRS pathogenesis. Overall, these data provide further support for current hypotheses of CRS and nasal polyposis pathogenesis, provide essential temporal context to studies on biomarkers and mechanisms of nasal polyposis, and highlight areas of focus for future targeted therapeutic options.

### 4.7 Summary and concluding remarks

This study presents a comprehensive multiomic time‐series analysis of CRS‐associated nasal polyp transcriptome, proteome, and microbiota, in three patients with CRSwNP. To our knowledge, this is the first study to investigate natural transcription and protein dynamics in CRS‐affected tissue over time.

High baseline interpatient variability and differential changes over time were detected in all metrics. Natural temporal variability was observed for a number of transcripts and proteins, including likely agents in the pathophysiology of CRS and nasal polyposis. Several markers that varied naturally over time have been previously identified as putative biomarkers of CRS, including *MUC5B, MUC5AC*, S100 calcium‐binding proteins, *CST1, EPX, CLC, POSTN*, and *CXCL8* (IL‐8). Several markers central to inflammation mediated by chemokine and cytokine, cadherin, integrin, Wnt, cytoskeletal, coagulation, and apoptosis signaling pathways were abundant in nasal polyp tissue at baseline. These processes were also often associated with markers that naturally changed over time, and those that responded to corticosteroid treatment.

These findings offer promising avenues for future research and candidate targets for the development of novel therapeutics addressing the pathophysiology of nasal polyposis.

## AUTHOR CONTRIBUTIONS

Michael Hoggard contributed to study design, sample processing, data acquisition, data analyses, and writing of the manuscript. Melissa Zoing and Richard G. Douglas contributed to subject recruitment and sampling. Bincy Jacob and Martin Middleditch contributed to proteomic analysis and data processing. David Wheeler contributed to transcriptomic data processing and analysis. Kevin Chang contributed to statistical analyses. Michael W. Taylor, Richard G. Douglas, and Kristi Biswas contributed to study design and provided laboratory space and materials. All authors contributed to the editing of the manuscript.

## ETHICS STATEMENT

This study was approved by the New Zealand Health and Disability Ethics Committee (14/NTA/134), and written informed consent was obtained from all participants.

## Supporting information

Supporting information.

Supporting information.

Supporting information.
