# Supplementary material for: Multiomic analysis identifies natural intrapatient temporal variability and changes in response to systemic corticosteroid therapy in chronic rhinosinusitis
Source: Immun Inflamm Dis. 2020 Nov 21;9(1):90–107. doi: 10.1002/iid3.349 (PMC7860613; doi:10.1002/iid3.349)
Supplement: Supplementary file 1 — Supporting information. [file IID3-9-90-s001.docx]

# Multiomic analysis identifies natural intra-patient temporal variability and changes in response to systemic corticosteroid therapy in chronic rhinosinusitis: Supplementary Methods

Michael Hoggard^a^, Bincy Jacob^a,b^, David Wheeler^c,1^, Melissa Zoing^d^, Kevin Chang^e^, Kristi Biswas^d^, Martin Middleditch^a,b^, Richard G. Douglas^d^, Michael W. Taylor^a,f^

^a^ School of Biological Sciences, The University of Auckland, Auckland, New Zealand.

^b^ Auckland Science Analytical Services, The University of Auckland, Auckland, New Zealand.

^c^ Nextgen Bioinformatic Services, Palmerston North, New Zealand.

^d^ School of Medicine, The University of Auckland, Auckland, New Zealand.

^e^ Department of Statistics, Statistical Consulting Centre, The University of Auckland,

Auckland, New Zealand.

^f^ Maurice Wilkins Centre for Molecular Biodiscovery, The University of Auckland, Auckland, New Zealand.

^1^ Present address: Department of Primary Industry, NSW, Australia.

# Supporting Information 1: Supplementary Methods

## Patient recruitment and sample collection

Three patients with CRSwNP listed for bilateral functional endoscopic sinus surgery for CRS were recruited. All patients were male, of New Zealand European ancestry, non-smokers, aged from 46 to 59 years, and with Lund-Mackay clinical severity scores ranging 17 to 23. None of the patients had taken antibiotics or corticosteroids in the four weeks prior to the study. CRSwNP diagnosis was made as per European position paper (EPOS2020) guidelines^E1^. Exclusion criteria included immunodeficiency, sinonasal vasculitis, and age < 18 years. This study was approved by the New Zealand Health and Disability Ethics committee (14/NTA/134), and written informed consent was obtained from all participants.

Samples were collected at three time points over two consecutive weeks (time points i, ii, and iii, including two sample collections in clinic, and a third at the time of surgery). Following the second clinic appointment, patients were prescribed oral prednisone (30 mg daily) for one week, followed by a final sample collection intraoperatively. Sample collection was designed to enable investigation of each of the following: **1.** **baseline inter-patient variability** (patients 1 vs. 2. vs. 3, incorporating both pre-treatment times [i + ii] to accommodate natural variability over time); **2. natural variability over time** (time points i vs. ii); and **3.** **treatment effects of corticosteroids** (prednisone) (pre-treatment time points vs. post-treatment [iii]). At each time point, symptom severity scores (SNOT-22^E2^) and two small adjacent nasal polyp tissue biopsies (< 0.1 g, ~1 mm diameter) were collected by an ORL surgeon (RGD) under endoscopic guidance, and placed into sterile 1.5 mL tubes on ice. For each time point, one biopsy, intended for human transcriptome (RNAseq) and microbiota analyses (gene-targeted amplicon sequencing), was placed into RNA*later* (Life Technologies, Auckland, New Zealand). The matched polyp biopsy, intended for human proteome analysis, was placed directly into an empty collection tube. All samples were stored at -20°C until the time of sample processing.

## Sample processing

Transcriptome and microbiota community sequencing. DNA and RNA were extracted in parallel from nasal polyp tissue biopsies collected in RNA*later* using the Qiagen AllPrep DNA/RNA isolation kit (Qiagen, Hilden, Germany). Biopsies were placed in sterile Lysing Matrix E bead tubes (MP Biomedicals, Sydney, Australia), together with 600 µL RLT lysis buffer (10 µL/mL β-mercaptoethanol added, as per kit recommendation), ruptured in a Tissue Lyser II (Qiagen, Hilden, Germany) for 2 x 40 s at 25 m/s, centrifuged, and the supernatant transferred to supplied spin columns and processed for DNA and RNA extraction as per the manufacturer’s instructions. Negative extractions (omitting any sample material) were conducted in parallel to test for contaminating microbial DNA. DNA and RNA quality and quantity were checked via NanoPhotometer (N60) (Implen GmbH, München, Germany).

Extracted RNA for transcriptome analysis (RNAseq) was treated with DNaseI (Amplification grade) (Life Technologies, Auckland, New Zealand) as per the manufacturer’s instructions, checked for residual DNA via β-actin PCR, and submitted to the sequencing provider (Auckland Genomics Ltd., Auckland, New Zealand) for final sample processing, library preparation, and sequencing on two lanes of an Illumina HiSeq machine (150 bp paired-end reads). Raw RNAseq sequence data have been uploaded to the SRA-NCBI repository (BioProject accession: PRJNA608823).

Extracted DNA was used for microbial community amplicon sequencing. Bacterial 16S rRNA and fungal ITS2 genomic markers were PCR amplified in triplicate using HotStar DNA polymerase (Qiagen, Hilden, Germany), ~100 to 200 ng gDNA template, and the primers 341F – 785R^E3^, and ITS3 – ITS4^E4^ (incorporating Nextera DNA Library Prep Kit Nextera), as previously described^E5-E6^. In brief, 16S amplicons were PCR amplified at 95°C (15 min), 35 cycles of 95°C (30 s), 55°C (30 s), and 70°C (40 s), and a final step of 70°C (3 min). ITS PCR conditions were as follows: 95°C (15 min), 35 cycles of 95°C (30 s), 52°C (30 s), and 70°C (60 s), and a final step of 70°C (7 min). Negative extraction control products and non-template PCR controls were included in each PCR, and checked via gel electrophoresis, with no detectable amplified product in any kit or PCR negative controls. Triplicate PCR products for each sample were pooled, purified using AMPure beads (Beckman-Coulter, Brea, CA), normalized to 3 ng/µL, and submitted to the sequencing provider (Auckland Genomics Ltd., Auckland, New Zealand) for library preparation and sequencing on the Illumina MiSeq platform (2 x 300 bp, paired-end reads). Microbiota raw sequence data have been uploaded to the SRA-NCBI repository (BioProject accession: PRJNA608821).

Proteome (SWATH-MS). Remaining polyp biopsies (those not collected in RNA*later*) were sonicated on ice in 150 µL urea/thiourea/dithiothreitol (DTT) buffer (7 M urea, 2 M thiourea, 10 mM DTT in 50 mM ammonium bicarbonate) using a Soniprep 150 sonicator (MSE, London, UK) for 4 x 30 s at ~10-15 microns. Disulphides were reduced via incubation at 56°C in a Discover chilled microwave (CEM Corp, Matthews, SC, USA) at 30 W power, cysteines alkylated by addition of 7.5 µL iodoacetamide (IAM), samples incubated in the dark at room temperature for 30 min, followed by addition of 3 µL 1M DTT to quench IAM. Total protein for each sample was quantified via EZQ assays (Life Technologies, Auckland, New Zealand) as per the manufacturer’s instructions, with results read on an Enspire plate reader (PerkinElmer, Waltham, MA, USA). Thirty micrograms of each sample was diluted 10-fold in 50 mM ammonium bicarbonate to permit trypsin digestion with 1 µg sequencing grade trypsin (Promega, Madison, WI, USA), and samples incubated at 45°C for 2 h in a chilled microwave at 15 W power. Samples were acidified to pH 3 via addition of 50% formic acid, centrifuged for 3 min at 16,000 *g*, and purified using 10 mg OASIS HLB SPE cartridges (Waters, Milford, MA, USA) as per the manufacturer’s instructions. Purified proteins were eluted in 300 µL 50% acetonitrile in 0.1% formic acid, then concentrated to a final volume of 20 µL via speedvac (ThermoSavant, Holbrook, NY, USA). A spectral library was constructed from a search of discovery tandem mass spectrometry (MS/MS) runs of pooled samples on a nanoLC-equipped TripleTOF 6600 mass spectrometer (Sciex, Framingham, MA, USA) using ProteinPilot v5 (Sciex, Singapore). LC-MS/MS was then conducted for each sample using SWATH acquisition, with fragment ion areas (and Benjamini and Hochberg False Discovery Rate [FDR] calculations for each ion) calculated by PeakView (v. 2.2) with the SWATH MicroApp 2.0 (Sciex, Singapore).

## Data processing

Transcriptome (RNAseq data)*.* Raw RNAseq reads (> 185 million/sample) were processed using BBDuk^E7^ to remove sequencing adapters and trim reads to a minimum phred quality score of 10. Any reads shorter than 50 bp following trimming were discarded. The high quality reads were mapped to the Ensembl Human genome (v. GRCh38)^E8^ using HISAT2^E9^ (> 90% of filtered reads successfully mapped). Read counts were allocated to the Ensembl human gene models (GRCh38.94) by HT-Seq (v. 0.6.0)^E10^ using the ‘Union mode’.

Proteome (SWATH-MS data). Data for each patient (three samples each) were processed independently using Excel. For each, fragment ion areas were filtered for type I error and for background noise (FDR adjusted *p*-value < 0.05; ion area > 3000). Samples for each patient were normalized, and fragments not present in all three time points, or with retention times < 25 min, were removed. Finally, sums of fragment areas for each peptide were calculated, followed by peptide area sums for each protein. Protein area sums were used for all subsequent analyses.

Microbiota (16S rRNA and ITS2 marker amplicon sequencing data). Bioinformatics processing of 16S rRNA gene and ITS2 marker amplicon sequences was conducted in USEARCH (v. 10)^E11-E14^, as previously described^E6, E15^. In brief, bacterial 16S rRNA gene amplicon sequences were merged, quality filtered, denoised, and zero-radius operational taxonomic units (ZOTUs; based on 100% sequence similarity, and analogous to amplicon sequence variants (ASV)) generated. Sequences were taxonomically assigned using the Silva living tree species project database (v. 123)^E16^, unassigned ZOTUs checked via NCBI blastn online, and non-target sequences (such as human reads) discarded. For fungal ITS2 marker sequences, reverse reads were discarded, forward reads truncated to 200 bp, and further trimmed to remove residual reverse primer binding regions (when present). Reads were filtered, denoised, ZOTUs generated, taxonomically assigned using the UNITE database (v. 22.08.2016)^E17^, unassigned reads manually checked via NCBI blastn, and non-target (e.g. human) reads discarded. Bacterial and fungal ZOTU tables were generated, subsampled to an even depth of 1300 sequences per sample, and alpha diversity indices (richness, Shannon diversity, and Simpson evenness) calculated in USEARCH. Full details of the bioinformatics processing are available online: https://github.com/mhog025/Microbiota-amplicon-bioinformatics.

## Data analyses and statistics

An initial exploratory analysis of natural transcript and protein variability over time was conducted by calculating ratios of normalized raw read counts between the first and second time points (times i and ii) for each individual patient (excluding transcripts with read counts < 1000, as these can misrepresent the scale of fold differences).

Transcriptome. The R package DESeq2 (v. 1.14.1)^E18, E19^ was used to identify differentially expressed genes (DEG) based on the read count data, as described in the package’s vignette. The following RNAseq comparisons were used: 1. natural variability (time point i vs. ii); and 2. response to treatment (time point ii vs. iii). Reported significant DEG are based on FDR adjusted *p*-values (α = 0.05) (DEG with unadjusted *p*-values < 0.05 are also presented in plots [clearly demarcated]). Ensembl IDs were also converted to HUGO Gene Nomenclature Committee (HGNC) symbols for ease of interpretation and to standardize reporting between RNAseq and proteome results.

Proteome. Log-transformed (natural log) data were tested for the following: 1. baseline inter-patient differences (time points i + ii for each patient); 2. natural variability (time point i vs. ii vs. iii); and 3. treatment effects (‘control’ times [i + ii] vs. ‘treatment’ [iii]). For inter-patient comparisons, linear models were fitted for each protein (response = log-transformed protein data; predictor = patient), and tested using one-way analysis of variance (ANOVA). For ‘natural variability’ and ‘treatment’ comparisons, linear mixed effects models were fitted with the addition of inter-patient differences fitted as random effects (to accommodate inter-patient differences prior to assessing the effects of time and treatment), and tested using ANOVA. For ‘inter-patient’ (patient 1 vs. 2 vs. 3) and ‘natural variability’ (times i vs. ii vs. iii), post-hoc testing of variables with ANOVA *p*-values < 0.05 was conducted via two-sample t-tests and Tukey’s honest significant difference tests (Tukey’s *p*-value α = 0.05). For ‘treatment’ (‘control’ vs ’treatment’), ANOVA results are reported (ANOVA *p*-value α = 0.05). UniProt IDs were also converted to HGNC symbols to standardize reporting between proteome and RNAseq results.

PANTHER analyses and pathways of interest data subset (‘pathways_subset’). Molecular pathways and gene ontology (GO) terms enriched for differentially expressed genes and proteins (DEG with FDR adjusted *p*-value < 0.1; proteins with Tukey’s *p*-value < 0.05) for each of the separate treatment comparisons (natural variability: times i vs. ii; and response to prednisone: times ii vs. iii) were identified using PANTHER (v. 14.0)^E20, E21^. Significant enrichment was based on PANTHER’s functional classification tool using the statistical overrepresentation test (release 2018-11-13) (FDR adjusted *p­-*value α = 0.05), using GO database release 2018-12-01^E22^.

To perform more focused sub-analyses investigating biologically relevant mechanisms likely to be involved in CRS processes, a subset of genes (898 genes) were identified based on their involvement in the following PANTHER pathways of interest (herein referred to as ‘pathways_subset’): inflammation mediated by chemokine and cytokine, interferon-gamma, interleukin, TGF-beta, T cell activation, B cell activation, Toll receptor, cadherin, Wnt, blood coagulation, and apoptosis signaling pathways. Transcript and protein data subsets were established for 813 pathways_subset-matching transcripts, and 66 pathways_subset-matching proteins, respectively.

Combined data. Remaining analyses were conducted in R (v. 3.3.0)^E18^ using log-transformed normalized read counts for each data set. Patient symptom scores, the 20 most abundant bacterial ZOTUs and fungal ZOTUS, and microbiota diversity indices were compared between: 1. patients; and 2. time points (i, ii, and iii) via Kruskal-Wallis tests with FDR adjustment, with post-hoc pairwise testing of significant variables via Dunn’s test of multiple comparisons (*p*-values) together with FDR adjustment (α = 0.05). Bray-Curtis dissimilarities were calculated for all data sets (full RNAseq, full proteome, pathways_subset RNAseq, pathways_subset proteome, bacterial community, and fungal community data) using the vegan package (v. 2.5-1)^E23^. Hierarchical clustering analyses and ordination analyses (nMDS, beta-dispersion, and adonis [permutations = 999 for each]) were conducted based on Bray-Curtis dissimilarities. Beta-dispersion analyses were calculated separately for each grouping of ‘patient’ and ‘treatment’ (pre-treatment vs. post-treatment) (none were significantly differently dispersed). Adonis incorporated patient differences first, followed by treatment.

Spearman correlation analyses were calculated for the following: 1. The 20 most abundant variables from each data set (transcriptome, proteome, bacterial ZOTUs, fungal ZOTUs); and 2. variables shared between transcriptome and proteome data sets based on matching HGNC IDs (proteins with incomplete data sets across all 9 samples were excluded). For the latter, correlation analyses were conducted for each matching data pair (comparing transcription data with its related protein), with a focus on negative correlation patterns. Correlation analyses included pairwise testing of significance via cor_pmat(), as well as FDR adjustment calculation (α = 0.05 in both cases).

Heat maps of log-transformed data were generated with hierarchical clustering of the variables. Transcriptome and proteome variables were divided into several clusters (depending on the number of variables) to simplify presentation of individual transcripts/proteins and associated PANTHER pathways. For visual clarity, figures are color-coded throughout as follows: Transcriptome data = red; proteome data = purple; bacterial data = green; fungal data = yellow.

# Supplementary Methods: References

E1. Fokkens WJ, Lund VJ, Hopkins C, Hellings PW, Kern R, Reitsma S, et al. European Position Paper on Rhinosinusitis and Nasal Polyps 2020. Rhinology. 2020;58:1–464.

E2. Hopkins C, Gillett S, Slack R, Lund VJ, Browne JP. Psychometric validity of the 22-item Sinonasal Outcome Test. Clin Otolaryngol. 2009;34:447–54.

E3. Herlemann DP, Labrenz M, Jürgens K, Bertilsson S, Waniek JJ, Andersson AF. Transitions in bacterial communities along the 2000 km salinity gradient of the Baltic Sea. ISME J. 2011;541:1571–9.

E4. White TJ, Bruns TD, Lee S, Taylor JW. Amplification and direct sequencing of fungal ribosomal RNA genes for phylogenetics. In: Innis MA, Gelfand DH, Sninsky JJ, White TJ, editors. PCR Protocols: A Guide to Methods and Applications. New York: Academic; 1990. p. 315–22.

E5. Hoggard M, Biswas K, Zoing M, Wagner Mackenzie B, Taylor MW, Douglas RG. Evidence of microbiota dysbiosis in chronic rhinosinusitis. Int Forum Allergy Rhinol. 2017;7:230–9.

E6. Hoggard M, Vesty A, Wong G, Montgomery JM, Fourie C, Douglas RG, et al. Characterizing the Human Mycobiota: A Comparison of Small Subunit rRNA, ITS1, ITS2, and Large Subunit rRNA Genomic Targets. Front Microbiol. 2018;9:2208.

E7. Bushnell B. Bbmap short read aligner. Berkeley, California: University of California; 2016.

E8. Zerbino DR, Achuthan P, Akanni W, Amode MR, Barrell D, Bhai J, et al. Ensembl 2018. Nucleic Acids Res. 2018;46:D754–61.

E9. Kim D, Langmead B, Salzberg SL. HISAT: A fast spliced aligner with low memory requirements. Nat Methods. 2015;12:357–60.

E10. Anders S, Pyl PT, Huber W. HTSeq-A Python framework to work with high-throughput sequencing data. Bioinformatics. 2015;31:166–9.

E11. Edgar RC. Search and clustering orders of magnitude faster than BLAST. Bioinformatics. 2010;26:2460–1.

E12. Edgar RC, Flyvbjerg H. Error filtering, pair assembly and error correction for next-generation sequencing reads. Bioinformatics. 2015;31:3476–82.

E13. Edgar RC. UNOISE2: improved error-correction for Illumina 16S and ITS amplicon sequencing. bioRxiv. 2016;

E14. Edgar RC. SINTAX: a simple non-Bayesian taxonomy classifier for 16S and ITS sequences. bioRxiv. 2016;

E15. Hoggard M, Zoing M, Biswas K, Taylor MW, Douglas RG. The sinonasal mycobiota in chronic rhinosinusitis and control patients. Rhinology. 2019;57:190–9.

E16. Yarza P, Richter M, Peplies J, Euzeby J, Amann R, Schleifer KH, et al. The All-Species Living Tree project: A 16S rRNA-based phylogenetic tree of all sequenced type strains. Syst Appl Microbiol. 2008;31:241–50.

E17. UNITE Community. UNITE USEARCH/UTAX release. UNITE Community; 2017. p. doi: 10.15156/BIO/587476.

E18. R Core Team. R: A language and environment for statistical computing. R Foundation for Statistical Computing. Vienna, Austria; 2015.

E19. Love MI, Huber W, Anders S. Moderated estimation of fold change and dispersion for RNA-seq data with DESeq2. Genome Biol. 2014;15:550.

E20. Mi H, Huang X, Muruganujan A, Tang H, Mills C, Kang D, et al. PANTHER version 11: Expanded annotation data from Gene Ontology and Reactome pathways, and data analysis tool enhancements. Nucleic Acids Res. 2017;45:D183–9.

E21. Mi H, Muruganujan A, Casagrande JT, Thomas PD. Large-scale gene function analysis with the panther classification system. Nat Protoc. 2013;8:1551–66.

E22. Carbon S, Dietze H, Lewis SE, Mungall CJ, Munoz-Torres MC, Basu S, et al. Expansion of the gene ontology knowledgebase and resources. Nucleic Acids Res. 2017;45:D331–8.

E23. Oksanen J, Blanchet FG, Kindt R, Legendre P, Minchin PR, O’Hara RB, et al. Vegan Community Ecology Package. R package version 2.3.2. 2015.
