# Supplementary material for: Multiomic analysis identifies natural intrapatient temporal variability and changes in response to systemic corticosteroid therapy in chronic rhinosinusitis
Source: Immun Inflamm Dis. 2020 Nov 21;9(1):90–107. doi: 10.1002/iid3.349 (PMC7860613; doi:10.1002/iid3.349)
Supplement: Supplementary file 2 — Supporting information. [file IID3-9-90-s002.docx]

| Supporting Information 2: Table S1. Patient information | | | |
| --- | --- | --- | --- |
|  | Patient 1 | Patient 2 | Patient 3 |
| Age | 46 | 54 | 59 |
| Gender | Male | Male | Male |
| Ethnicity | NZE | NZE | NZE |
| Smoker | N | N | N |
| Asthma | Y | N | N |
| Aspirin sensitivity | N | N | N |
| Antibiotics^a^ | N | N | N |
| Corticosteroids^a^ | N | N | N |
| SNOT22_visit1 | 33 | 26 | 36 |
| SNOT22_visit2 | 35 | 21 | 28 |
| SNOT22_visit3 | 29 | 18 | 26 |
| Lund-Mackay | 23 | 17 | 23 |
| ^a^ Whether patients had taken antibiotics or corticosteroids in the 4 weeks prior to surgery. NZE = New Zealand European ancestry. SNOT22 = Sinonasal outcome test symptom score. | | | |

| Supporting Information 2: Table S2. Baseline pathways_subset proteins: inter-patient pairwise comparisons | | | |
| --- | --- | --- | --- |
|  | **Contrast** | **Ratio** | ***p*-value** |
| P12724_(RNASE3)_(ribonuclease A family member 3) | 1 vs 2 | 45.3 | 0.006 |
| Q05707_(COL14A1)_(collagen type XIV alpha 1 chain) | 2 vs 3 | 26.4 | 0.002 |
| P35749_(MYH11)_(myosin heavy chain 11) | 2 vs 3 | 22.3 | 0.010 |
| Q05707_(COL14A1)_(collagen type XIV alpha 1 chain) | 1 vs 2 | -17.8 | 0.003 |
| P00488_(F13A1)_(coagulation factor XIII A chain) | 1 vs 2 | 12.0 | 0.017 |
| P00488_(F13A1)_(coagulation factor XIII A chain) | 1 vs 3 | 11.6 | 0.018 |
| O15143_(ARPC1B)_(actin related protein 2/3 complex subunit 1B) | 1 vs 2 | 9.4 | 0.009 |
| P01042_(KNG1)_(kininogen 1) | 1 vs 3 | -8.6 | 0.009 |
| P35749_(MYH11)_(myosin heavy chain 11) | 1 vs 2 | -6.5 | 0.039 |
| C9JFR7_(CYCS)_(cytochrome c, somatic) | 1 vs 2 | 6.2 | 0.042 |
| P06744_(GPI)_(glucose-6-phosphate isomerase) | 1 vs 3 | 6.1 | 0.003 |
| P01042_(KNG1)_(kininogen 1) | 1 vs 2 | -5.3 | 0.018 |
| P16050_(ALOX15)_(arachidonate 15-lipoxygenase) | 1 vs 3 | 5.2 | 0.032 |
| P00747_(PLG)_(plasminogen) | 1 vs 2 | -4.6 | 0.006 |
| P16050_(ALOX15)_(arachidonate 15-lipoxygenase) | 1 vs 2 | 4.1 | 0.048 |
| P62736_(ACTA2)_(actin, alpha 2, smooth muscle, aorta) | 1 vs 3 | 4.1 | 0.008 |
| P62736_(ACTA2)_(actin, alpha 2, smooth muscle, aorta) | 2 vs 3 | 4.0 | 0.009 |
| O15144_(ARPC2)_(actin related protein 2/3 complex subunit 2) | 1 vs 3 | 4.0 | 0.003 |
| P04899_(GNAI2)_(G protein subunit alpha i2) | 1 vs 3 | 4.0 | 0.001 |
| P00747_(PLG)_(plasminogen) | 2 vs 3 | 3.9 | 0.009 |
| P35579_(MYH9)_(myosin heavy chain 9) | 1 vs 3 | 3.8 | < 0.001 |
| P35221_(CTNNA1)_(catenin alpha 1) | 1 vs 3 | 3.7 | 0.037 |
| P63000_(RAC1)_(Rac family small GTPase 1) | 1 vs 3 | 3.7 | 0.013 |
| P61160_(ACTR2)_(ARP2 actin related protein 2 homolog) | 1 vs 3 | 3.4 | 0.006 |
| P11021_(HSPA5)_(heat shock protein family A (Hsp70) member 5) | 1 vs 3 | 3.3 | 0.032 |
| P61586_(RHOA)_(ras homolog family member A) | 1 vs 3 | 3.3 | 0.046 |
| P61586_(RHOA)_(ras homolog family member A) | 1 vs 3 | 3.3 | 0.046 |
| P06744_(GPI)_(glucose-6-phosphate isomerase) | 1 vs 2 | 3.3 | 0.011 |
| O15511_(ARPC5)_(actin related protein 2/3 complex subunit 5) | 1 vs 3 | 3.2 | 0.016 |
| A0A087X0S5_(COL6A1)_(collagen type VI alpha 1 chain) | 1 vs 2 | -2.9 | 0.042 |
| O15145_(ARPC3)_(actin related protein 2/3 complex subunit 3) | 1 vs 3 | 2.9 | 0.008 |
| P11021_(HSPA5)_(heat shock protein family A (Hsp70) member 5) | 1 vs 2 | 2.8 | 0.049 |
| Q5JR08_(RHOC)_(ras homolog family member C) | 1 vs 2 | 2.4 | 0.022 |
| Q5JR08_(RHOC)_(ras homolog family member C) | 1 vs 2 | 2.4 | 0.022 |
| P60709_(ACTB)_(actin beta) | 1 vs 3 | 2.3 | 0.022 |
| Q9BPX5_(ARPC5L)_(actin related protein 2/3 complex subunit 5 like) | 1 vs 3 | 2.3 | 0.006 |
| P04899_(GNAI2)_(G protein subunit alpha i2) | 1 vs 2 | 2.3 | 0.005 |
| O15144_(ARPC2)_(actin related protein 2/3 complex subunit 2) | 2 vs 3 | 2.1 | 0.018 |
| P35579_(MYH9)_(myosin heavy chain 9) | 2 vs 3 | 2.1 | 0.001 |
| P61769_(B2M)_(beta-2-microglobulin) | 1 vs 3 | 2.1 | 0.049 |
| P61160_(ACTR2)_(ARP2 actin related protein 2 homolog) | 1 vs 2 | 2.0 | 0.029 |
| O15145_(ARPC3)_(actin related protein 2/3 complex subunit 3) | 2 vs 3 | 1.9 | 0.032 |
| O15144_(ARPC2)_(actin related protein 2/3 complex subunit 2) | 1 vs 2 | 1.9 | 0.030 |
| P35579_(MYH9)_(myosin heavy chain 9) | 1 vs 2 | 1.8 | 0.002 |
| P04899_(GNAI2)_(G protein subunit alpha i2) | 2 vs 3 | 1.8 | 0.015 |
| * Included proteins are from the subset that that matched hits from the PANTHER pathways of interest as outlined in this study (pathways_subset). Linear models were fitted for each protein and tested via ANOVA, with post-hoc pairwise testing via two-sample t-tests and Tukey's honest significant difference testing. *P*-value = Tukey's *p*-value. All significant pairwise comparisons are presented. | | | |

| Supporting Information 2: Table S3. Natural intra-patient transcriptome and proteome variability* | | | |
| --- | --- | --- | --- |
|  | **Ratio difference (time i vs. ii)** | | |
|  | **Patient_1** | **Patient_2** | **Patient_3** |
| Transcriptome (pathways_subset) |  |  |  |
| ENSG00000129538_(RNASE1) | 3.0 | -24.4 | -4.3 |
| ENSG00000169429_(CXCL8) | -3.3 | 13.4 | -4.6 |
| ENSG00000175899_(A2M) | 1.3 | -9.3 | -6.3 |
| ENSG00000142156_(COL6A1) | -1.4 | -11.4 | -1.9 |
| ENSG00000175592_(FOSL1) | -7.5 |  | 2.0 |
| ENSG00000275385_(CCL18) | -3.1 | -7.3 | -1.7 |
| ENSG00000110799_(VWF) | 1.4 |  | -6.2 |
| ENSG00000142173_(COL6A2) | -3.0 | -3.3 | -4.3 |
| ENSG00000130513_(GDF15) | -2.0 | 5.4 | 1.9 |
| ENSG00000184557_(SOCS3) | 1.1 |  | -5.1 |
| ENSG00000181085_(MAPK15) | 3.6 | 3.6 | 1.6 |
| ENSG00000118503_(TNFAIP3) | -1.9 | 4.1 | -2.4 |
| ENSG00000170458_(CD14) | -1.7 | -1.0 | -4.9 |
| ENSG00000162552_(WNT4) | 3.2 | 1.9 | 2.1 |
| ENSG00000122861_(PLAU) | -4.2 | 1.8 | 1.1 |
| ENSG00000115415_(STAT1) | -2.2 | 3.5 | 1.3 |
| ENSG00000102908_(NFAT5) | -1.3 | -3.6 | -1.8 |
| ENSG00000181104_(F2R) | -1.0 | -2.0 | 3.7 |
| ENSG00000006210_(CX3CL1) | 2.4 | -3.1 | 1.1 |
| ENSG00000271503_(CCL5) | -1.0 | 3.1 | -2.2 |
| ENSG00000083857_(FAT1) | 1.1 | -3.9 | 1.1 |
| ENSG00000011422_(PLAUR) | -3.6 | 1.2 | -1.1 |
| Proteome |  |  |  |
| P59665_(DEFA1)_(defensin alpha 1 (HGNC:2761)) | 9.5 |  |  |
| P49207_(RPL34)_(ribosomal protein L34 (HGNC:10340)) | 1.6 |  | 13.8 |
| P02533_(KRT14)_(keratin 14 (HGNC:6416)) | -6.8 |  |  |
| P62857_(RPS28)_(ribosomal protein S28 (HGNC:10418)) | -5.6 |  |  |
| P02675_(FGB)_(fibrinogen beta chain (HGNC:3662)) | -1.0 | -13.9 | -1.8 |
| P05109_(S100A8)_(S100 calcium binding protein A8 (HGNC:10498)) | 1.0 | -12.2 | -2.2 |
| P02671_(FGA)_(fibrinogen alpha chain (HGNC:3661)) | -1.4 | -9.6 | -1.8 |
| P02679_(FGG)_(fibrinogen gamma chain (HGNC:3694)) | -1.6 | -9.1 | -2.0 |
| B0YIW2_(APOC3)_(apolipoprotein C3 (HGNC:610)) | -1.2 | -6.3 | -4.9 |
| P00915_(CA1)_(carbonic anhydrase 1 (HGNC:1368)) | -8.7 | 2.1 | 1.4 |
| P11678_(EPX)_(eosinophil peroxidase (HGNC:3423)) | -1.0 | -1.1 | 9.2 |
| P06702_(S100A9)_(S100 calcium binding protein A9 (HGNC:10499)) | 1.2 | -7.8 | -2.2 |
| P02042_(HBD)_(hemoglobin subunit delta (HGNC:4829)) | -7.4 | 1.7 | 1.1 |
| Q05315_(CLC)_(Charcot-Leyden crystal galectin (HGNC:2014)) | 1.1 | 2.2 | 6.7 |
| P01037_(CST1)_(cystatin SN (HGNC:2473)) | -1.2 | 5.4 |  |
| P30838_(ALDH3A1)_(aldehyde dehydrogenase 3 family member A1 (HGNC:405)) | -2.4 | 6.2 | 1.1 |
| P69905_(HBA1)_(hemoglobin subunit alpha 1 (HGNC:4823)) | -6.5 | 1.8 | 1.1 |
| Q15063_(POSTN)_(periostin (HGNC:16953)) | 1.0 | 7.3 | 1.1 |
| D6RGG3_(COL12A1)_(collagen type XII alpha 1 chain (HGNC:2188)) | -1.5 | 5.7 | 1.9 |
| Q8IUX7_(AEBP1)_(AE binding protein 1 (HGNC:303)) | -1.2 | 5.4 | 2.4 |
| P68871_(HBB)_(hemoglobin subunit beta (HGNC:4827)) | -5.9 | 1.5 | 1.1 |
| P08311_(CTSG)_(cathepsin G (HGNC:2532)) | -1.0 | -5.3 | -1.8 |
| A0A0C4DH31_(IGHV1-18)_(immunoglobulin heavy variable 1-18 (HGNC:5549)) | 1.1 | -5.4 | 1.0 |
| P13727_(PRG2)_(proteoglycan 2, pro eosinophil major basic protein (HGNC:9362)) | 1.2 | -1.1 | 5.0 |
| Transcriptome (full data set) |  |  |  |
| ENSG00000169474_(SPRR1A) | -52.6 |  |  |
| ENSG00000205420_(KRT6A) | -77.0 |  | 3.1 |
| ENSG00000170373_(CST1) | 3.0 | -109.2 | 6.0 |
| ENSG00000169469_(SPRR1B) | -43.8 |  | 3.7 |
| ENSG00000134757_(DSG3) | -18.5 |  |  |
| ENSG00000134827_(TCN1) | 1.6 | -41.0 | -5.1 |
| ENSG00000240583_(AQP1) | 3.1 | -27.6 | -14.5 |
| ENSG00000117983_(MUC5B) | 3.7 |  | -23.4 |
| ENSG00000163209_(SPRR3) | -17.0 |  | 4.9 |
| ENSG00000129538_(RNASE1) | 3.0 | -24.4 | -4.3 |
| ENSG00000188817_(SNTN) |  | 18.9 | -1.7 |
| ENSG00000167656_(LY6D) | -17.2 |  | 2.6 |
| ENSG00000196611_(MMP1) | -9.2 |  |  |
| ENSG00000147689_(FAM83A) | -8.8 |  |  |
| ENSG00000012223_(LTF) | 1.9 | -15.3 |  |
| ENSG00000173947_(PIFO) |  | 15.2 | -1.6 |
| ENSG00000124466_(LYPD3) | -18.3 | 1.2 | 5.1 |
| ENSG00000211897_(Unclassified) | 7.5 |  |  |
| ENSG00000004838_(ZMYND10) |  | 13.2 | -1.5 |
| ENSG00000065618_(Unclassified) | -14.9 | 1.5 | 4.8 |
| ENSG00000241794_(SPRR2A) |  |  | 7.0 |
| ENSG00000134531_(EMP1) | -17.9 | -1.2 | 1.6 |
| ENSG00000186847_(KRT14) |  |  | 6.9 |
| ENSG00000211895_(Unclassified) | 6.7 | -9.9 | -3.6 |
| ENSG00000163739_(CXCL1) | -2.5 | -1.3 | -16.4 |
| ENSG00000197253_(TPSB2) | 6.2 | -9.2 | -4.4 |
| ENSG00000196169_(KIF19) |  | 11.5 | -1.4 |
| ENSG00000179094_(Unclassified) | 4.8 | 2.7 | 11.2 |
| ENSG00000145491_(ROPN1L) |  | 10.8 | -1.7 |
| ENSG00000198840_(ND3) | 2.3 | -15.1 | 1.3 |
| ENSG00000211592_(Unclassified) | 3.8 | -12.7 | -2.0 |
| ENSG00000204950_(LRRC10B) |  | 11.3 | -1.1 |
| ENSG00000211890_(Unclassified) | 10.6 |  | -1.7 |
| ENSG00000178965_(ERICH3) |  | 10.3 | -2.0 |
| ENSG00000203985_(LDLRAD1) |  | 10.7 | -1.6 |
| ENSG00000157765_(SLC34A2) | 1.3 |  | -10.8 |
| ENSG00000172236_(TPSAB1) | 5.7 | -10.4 | -1.7 |
| ENSG00000126861_(Unclassified) | 3.2 | 12.3 | -1.9 |
| ENSG00000162004_(CCDC78) |  | 10.4 | -1.0 |
| ENSG00000162543_(UBXN10) |  | 9.1 | -2.2 |
| ENSG00000128510_(CPA4) | -5.7 |  |  |
| ENSG00000188931_(CFAP126) |  | 9.5 | -1.8 |
| ENSG00000010626_(LRRC23) | 2.7 | 13.3 | -1.0 |
| ENSG00000158486_(DNAH3) |  | 9.8 | -1.5 |
| ENSG00000077327_(SPAG6) |  | 9.5 | -1.8 |
| ENSG00000111834_(RSPH4A) |  | 9.5 | -1.8 |
| ENSG00000175899_(A2M) | 1.3 | -9.3 | -6.3 |
| ENSG00000198788_(Unclassified) | 5.5 |  |  |
| ENSG00000136918_(WDR38) |  | 9.7 | -1.1 |
| ENSG00000163751_(CPA3) | 2.9 | -9.9 | -3.3 |
| ENSG00000186191_(BPIFB4) | 5.3 |  |  |
| ENSG00000211896_(Unclassified) | 2.8 | -11.2 | -1.7 |
| ENSG00000168542_(Unclassified) | -7.6 | 1.1 | -6.7 |
| ENSG00000137975_(CLCA2) | -7.5 |  | 2.7 |
| ENSG00000211445_(GPX3) | 2.4 | -11.5 | -1.3 |
| ENSG00000211677_(Unclassified) | 4.6 | -5.5 |  |
| ENSG00000048342_(CC2D2A) |  | 8.1 | -2.1 |
| ENSG00000206075_(SERPINB5) | -10.4 | 2.0 | 2.7 |
| ENSG00000179813_(FAM216B) | 2.3 | 11.2 | -1.4 |
| ENSG00000277639_(LOC105371267) | 3.0 | 9.8 | -2.1 |
| ENSG00000275395_(FCGBP) | 6.1 | -6.0 | 2.7 |
| ENSG00000167858_(TEKT1) |  | 8.1 | -1.7 |
| ENSG00000142156_(COL6A1) | -1.4 | -11.4 | -1.9 |
| ENSG00000005448_(WDR54) |  | 8.2 | -1.5 |
| ENSG00000215217_(C5orf49) |  | 8.4 | -1.3 |
| ENSG00000170477_(KRT4) | -5.7 | 7.0 | 1.9 |
| ENSG00000114473_(IQCG) |  | 8.0 | -1.7 |
| ENSG00000173706_(HEG1) | 1.4 | -9.9 | -3.2 |
| ENSG00000166670_(MMP10) | 1.8 | -2.5 | -10.1 |
| ENSG00000198886_(ND4) | 1.8 | -11.5 | 1.1 |
| ENSG00000141294_(LRRC46) |  | 8.1 | -1.4 |
| ENSG00000188659_(SAXO2) |  | 8.0 | -1.6 |
| ENSG00000120262_(CCDC170) |  | 8.1 | -1.4 |
| ENSG00000215182_(MUC5AC) | 11.3 | -1.2 | 1.8 |
| ENSG00000175592_(FOSL1) | -7.5 |  | 2.0 |
| ENSG00000205084_(TMEM231) |  | 8.3 | -1.1 |
| ENSG00000159713_(TPPP3) | 3.3 | 9.5 | -1.2 |
| ENSG00000149021_(SCGB1A1) | 1.6 | -5.3 | -7.0 |
| ENSG00000135919_(SERPINE2) | -8.9 | -1.1 | 3.8 |
| ENSG00000118849_(RARRES1) | 2.1 | -2.0 | -9.8 |
| ENSG00000026025_(VIM) | -1.6 | -6.0 | -6.1 |
| ENSG00000101335_(MYL9) | 4.9 | -5.5 | -3.3 |
| ENSG00000198763_(ND2) | 1.4 | -10.8 | 1.4 |
| ENSG00000124237_(C20orf85) | 3.3 | 9.1 | -1.1 |
| ENSG00000163040_(CCDC74A) |  | 8.0 | -1.1 |
| ENSG00000007174_(DNAH9) |  | 7.1 | -1.9 |
| ENSG00000243955_(GSTA1) |  | 6.8 | 2.1 |
| ENSG00000152763_(WDR78) |  | 6.8 | -2.1 |
| ENSG00000198899_(ATP6) | 1.4 | -10.6 | 1.2 |
| ENSG00000164692_(COL1A2) | -5.9 | -1.1 | -6.1 |
| ENSG00000164972_(C9orf24) |  | 7.7 | -1.1 |
| ENSG00000129654_(FOXJ1) | 3.7 | 7.8 | -1.4 |
| ENSG00000233913_(Unclassified) | 1.8 | -1.2 | 9.9 |
| ENSG00000182853_(VMO1) | 2.6 | -8.7 | 1.5 |
| ENSG00000224383_(PRR29) |  | 7.0 | -1.5 |
| ENSG00000103021_(CCDC113) | 1.3 | 8.9 | -2.5 |
| ENSG00000090382_(LYZ) | 1.3 | -8.0 | -3.3 |
| ENSG00000172578_(KLHL6) |  | 6.5 | -1.8 |
| ENSG00000198183_(BPIFA1) | 4.8 | 1.8 | -6.0 |
| ENSG00000064300_(NGFR) | 1.9 | -8.4 | 2.3 |
| ENSG00000158023_(WDR66) |  | 7.1 | -1.2 |
| ENSG00000100228_(RAB36) |  | 5.8 | -2.5 |
| ENSG00000272398_(CD24) | -2.8 | 7.9 | -1.7 |
| ENSG00000166963_(MAP1A) |  | 5.8 | -2.4 |
| ENSG00000133265_(HSPBP1) | 1.5 | 9.7 | -1.0 |
| ENSG00000115112_(TFCP2L1) | 5.1 | 4.7 | 2.4 |
| ENSG00000113140_(SPARC) | -2.7 | -4.3 | -5.2 |
| ENSG00000138002_(IFT172) |  | 6.5 | -1.6 |
| ENSG00000133110_(POSTN) | -2.8 | -6.4 | 2.8 |
| ENSG00000128536_(CDHR3) |  | 6.6 | -1.4 |
| ENSG00000181378_(CFAP65) |  | 6.5 | -1.5 |
| ENSG00000171962_(DRC3) |  | 6.8 | 1.0 |
| ENSG00000137033_(IL33) | -1.4 | -7.5 | -2.9 |
| ENSG00000157514_(TSC22D3) | 3.1 | 1.3 | 7.3 |
| ENSG00000198786_(ND5) | 1.5 | -9.0 | 1.0 |
| ENSG00000198727_(CYTB) | 1.4 | -8.9 | 1.2 |
| ENSG00000128581_(IFT22) |  | 6.2 | -1.5 |
| ENSG00000124107_(SLPI) | 2.3 | -6.9 | -2.3 |
| ENSG00000248527_(Unclassified) | 1.8 | -8.3 | 1.4 |
| ENSG00000110799_(VWF) | 1.4 |  | -6.2 |
| ENSG00000108821_(COL1A1) | -6.3 | 1.6 | -3.5 |
| ENSG00000189334_(S100A14) | -5.4 | 2.1 | 3.9 |
| ENSG00000165698_(SPACA9) |  | 6.2 | -1.3 |
| ENSG00000114391_(RPL24) | -4.6 | 5.4 | 1.3 |
| ENSG00000258752_(Unclassified) |  | 6.3 | -1.2 |
| ENSG00000111912_(NCOA7) | -1.7 | 1.8 | -7.6 |
| ENSG00000198712_(COX2) | 1.6 | -7.9 | 1.5 |
| ENSG00000165474_(GJB2) | -8.9 | 1.0 | 1.0 |
| ENSG00000105519_(CAPS) | 3.7 | 6.1 | 1.1 |
| ENSG00000198938_(COX3) | 1.3 | -8.2 | 1.4 |
| ENSG00000237973_(Unclassified) | 1.6 | -6.6 | 2.7 |
| ENSG00000105388_(CEACAM5) | -1.4 | 1.5 | 7.9 |
| ENSG00000121898_(CPXM2) | 1.9 | -6.5 | -2.5 |
| ENSG00000151632_(AKR1C2) | -2.2 | 1.4 | 7.2 |
| ENSG00000182492_(BGN) | -1.3 |  | -6.0 |
| ENSG00000130300_(PLVAP) | 1.2 |  | -5.9 |
| ENSG00000197838_(CYP2A13) | 3.2 | -5.2 | -2.2 |
| ENSG00000125730_(C3) | 5.8 | -2.6 | -2.3 |
| ENSG00000263639_(MSMB) | 4.0 | -5.2 | 1.4 |
| ENSG00000087903_(RFX2) | 2.0 | 7.0 | -1.6 |
| ENSG00000128594_(LRRC4) | -3.1 | 5.8 | 1.6 |
| ENSG00000168658_(VWA3B) |  | 5.7 | -1.2 |
| ENSG00000133636_(NTS) | -1.2 | -6.1 | 3.1 |
| ENSG00000198804_(COX1) | 1.5 | -7.8 | 1.1 |
| ENSG00000134762_(DSC3) | -5.1 |  | 1.8 |
| ENSG00000135205_(CCDC146) |  | 5.5 | -1.4 |
| ENSG00000159588_(CCDC17) | 2.6 | 6.7 | 1.0 |
| ENSG00000096060_(FKBP5) | 1.8 |  | 5.0 |
| ENSG00000117114_(ADGRL2) | -1.4 | -6.2 | -2.6 |
| ENSG00000198848_(CES1) | 5.1 | 2.2 | -2.8 |
| ENSG00000198888_(ND1) | 1.2 | -7.2 | 1.7 |
| ENSG00000126368_(NR1D1) | 4.0 | -1.1 | 5.1 |
| ENSG00000160345_(C9orf116) |  | 5.4 | -1.2 |
| ENSG00000229119_(Unclassified) | 2.2 | -5.9 | 1.8 |
| ENSG00000091136_(LAMB1) | -1.3 | -7.0 | -1.6 |
| ENSG00000154099_(DNAAF1) |  | 5.4 | 1.2 |
| ENSG00000197641_(SERPINB13) | -6.0 | 1.0 | 2.7 |
| ENSG00000167552_(TUBA1A) | 2.0 | 6.5 | -1.2 |
| ENSG00000196754_(S100A2) | -5.3 | 1.2 | 3.0 |
| ENSG00000175164_(ABO) | -1.2 | -3.0 | 5.3 |
| ENSG00000185361_(TNFAIP8L1) | 2.1 | 6.0 | -1.4 |
| ENSG00000054690_(PLEKHH1) | 5.1 | -3.1 | 1.4 |
| ENSG00000122735_(DNAI1) |  | 5.2 | -1.1 |
| ENSG00000105974_(CAV1) | -5.2 |  | 1.1 |
| ENSG00000121653_(MAPK8IP1) | 2.3 | 5.9 | 1.1 |
| ENSG00000130513_(GDF15) | -2.0 | 5.4 | 1.9 |
| ENSG00000184557_(SOCS3) | 1.1 |  | -5.1 |
| ENSG00000162551_(ALPL) | 1.7 | -1.6 | 6.0 |
| ENSG00000112096_(Unclassified) | -2.6 | 1.3 | -5.4 |
| ENSG00000074410_(CA12) | -1.3 | -5.8 | 2.0 |
| ENSG00000100124_(Unclassified) | 2.0 | 5.8 | -1.1 |
| ENSG00000256612_(CYP2B7P) | 6.6 | 1.1 | -1.0 |
| ENSG00000119333_(WDR34) | 1.5 | 6.2 | 1.0 |
| ENSG00000131620_(ANO1) | -5.3 | 1.8 | 1.6 |
| ENSG00000108602_(ALDH3A1) | -1.3 | 1.5 | 5.8 |
| ENSG00000212907_(ND4L) | 1.0 | -6.4 | 1.1 |
| ENSG00000163071_(SPATA18) | 1.4 | 5.3 | -1.8 |
| ENSG00000103534_(TMC5) | 1.4 | 5.3 | -1.7 |
| ENSG00000174564_(IL20RB) | 1.3 | -5.2 | 1.9 |
| ENSG00000076706_(Unclassified) | -1.8 | -5.5 | -1.0 |
| ENSG00000163131_(CTSS) | -1.1 | 5.6 | -1.5 |
| ENSG00000106012_(IQCE) | 1.2 | 5.5 | -1.5 |
| ENSG00000117602_(RCAN3) | 1.2 | 5.6 | -1.3 |
| ENSG00000140403_(DNAJA4) | -1.0 | 5.1 | -1.8 |
| ENSG00000115758_(ODC1) | -5.2 | 1.5 | -1.1 |
| ENSG00000146425_(DYNLT1) | 1.0 | 5.7 | -1.0 |
| ENSG00000188229_(TUBB4B) | -1.1 | 5.5 | 1.1 |
| ENSG00000176903_(PNMA1) | 1.4 | 5.2 | -1.0 |
| ENSG00000168209_(DDIT4) | 1.5 | -1.1 | 5.0 |
| ENSG00000120306_(CYSTM1) | -1.1 | 5.3 | -1.2 |
| ENSG00000185215_(TNFAIP2) | 1.2 | -1.0 | -5.1 |
| * Ratios compare the difference between time point i and time point ii for each patient individually. Transcriptome variables from the pathways_subset data set with greater than three times difference within one or more patients, proteins with greater than five times difference, and transcriptome variables from the full data set with greater than five times difference, are presented. Missing data reflect transcript comparisons where at least one of the normalised transcript counts was less than 1000 (which were excluded from this analysis), or where proteins which were not detected in all three time points for that respective patient (and which were excluded from the data). | | | |

| Supporting Information 2: Table S4. Natural temporal variability: Proteins and Transcripts that differed significantly between times i and ii | | |
| --- | --- | --- |
|  | ***p*-value*** | ***p*-adjusted**** |
| Proteome |  |  |
| P01023(A2M) | 0.011 | 0.011 |
| P39060(COL18A1) | 0.014 | 0.014 |
| H0YI09(METTL7A) | 0.016 | 0.016 |
| P02649(APOE) | 0.027 | 0.027 |
| Q15393(SF3B3) | 0.043 | 0.043 |
| P50570(DNM2) | 0.047 | 0.047 |
| B7ZKJ8(ITIH4) | 0.048 | 0.048 |
| Transcriptome |  |  |
| ENSG00000078898 (BPIFB2) | < 0.001 | < 0.001 |
| ENSG00000109208 (SMR3A) | < 0.001 | < 0.001 |
| ENSG00000167916 (KRT24) | < 0.001 | < 0.001 |
| ENSG00000172236 (TPSAB1) | < 0.001 | < 0.001 |
| ENSG00000206172 (HBA1) | < 0.001 | < 0.001 |
| ENSG00000263628 (MIR3155A) | < 0.001 | < 0.001 |
| ENSG00000285624 (Unclassified) | < 0.001 | < 0.001 |
| ENSG00000221971 (Unclassified) | < 0.001 | < 0.001 |
| ENSG00000160862 (AZGP1) | < 0.001 | < 0.001 |
| ENSG00000056736 (IL17RB) | < 0.001 | < 0.001 |
| ENSG00000270571 (Unclassified) | < 0.001 | < 0.001 |
| ENSG00000244734 (HBB) | < 0.001 | < 0.001 |
| ENSG00000197744 (Unclassified) | < 0.001 | < 0.001 |
| ENSG00000188536 (HBA2) | < 0.001 | < 0.001 |
| ENSG00000102010 (BMX) | < 0.001 | < 0.001 |
| ENSG00000168481 (LGI3) | < 0.001 | < 0.001 |
| ENSG00000132821 (VSTM2L) | < 0.001 | < 0.001 |
| ENSG00000124440 (HIF3A) | < 0.001 | < 0.001 |
| ENSG00000168269 (FOXI1) | < 0.001 | < 0.001 |
| ENSG00000187908 (DMBT1) | < 0.001 | < 0.001 |
| ENSG00000198576 (ARC) | < 0.001 | < 0.001 |
| ENSG00000126549 (STATH) | < 0.001 | < 0.001 |
| ENSG00000232940 (HCG25) | < 0.001 | < 0.001 |
| ENSG00000285820 (Unclassified) | < 0.001 | < 0.001 |
| ENSG00000183476 (SH2D7) | < 0.001 | < 0.001 |
| ENSG00000165029 (ABCA1) | < 0.001 | < 0.001 |
| ENSG00000117707 (PROX1) | < 0.001 | < 0.001 |
| ENSG00000184905 (TCEAL2) | < 0.001 | < 0.001 |
| ENSG00000233967 (Unclassified) | < 0.001 | < 0.001 |
| ENSG00000251655 (PRB1) | < 0.001 | < 0.001 |
| ENSG00000268101 (Unclassified) | < 0.001 | < 0.001 |
| ENSG00000187398 (LUZP2) | < 0.001 | < 0.001 |
| ENSG00000099194 (SCD) | < 0.001 | < 0.001 |
| ENSG00000157680 (DGKI) | < 0.001 | < 0.001 |
| ENSG00000007216 (SLC13A2) | < 0.001 | 0.001 |
| ENSG00000171401 (KRT13) | < 0.001 | 0.001 |
| ENSG00000119125 (GDA) | < 0.001 | 0.001 |
| ENSG00000171201 (SMR3B) | < 0.001 | 0.001 |
| ENSG00000137077 (CCL21) | < 0.001 | 0.001 |
| ENSG00000105696 (TMEM59L) | < 0.001 | 0.001 |
| ENSG00000169474 (SPRR1A) | < 0.001 | 0.002 |
| ENSG00000177098 (SCN4B) | < 0.001 | 0.002 |
| ENSG00000135097 (MSI1) | < 0.001 | 0.002 |
| ENSG00000176009 (ASCL3) | < 0.001 | 0.002 |
| ENSG00000072310 (SREBF1) | < 0.001 | 0.003 |
| ENSG00000163209 (SPRR3) | < 0.001 | 0.003 |
| ENSG00000163017 (ACTG2) | < 0.001 | 0.003 |
| ENSG00000188175 (HEPACAM2) | < 0.001 | 0.003 |
| ENSG00000197253 (TPSB2) | < 0.001 | 0.003 |
| ENSG00000125999 (BPIFB1) | < 0.001 | 0.004 |
| ENSG00000063127 (SLC6A16) | < 0.001 | 0.004 |
| ENSG00000168952 (STXBP6) | < 0.001 | 0.005 |
| ENSG00000168309 (FAM107A) | < 0.001 | 0.005 |
| ENSG00000226360 (Unclassified) | < 0.001 | 0.005 |
| ENSG00000120885 (Unclassified) | < 0.001 | 0.005 |
| ENSG00000183876 (ARSI) | < 0.001 | 0.005 |
| ENSG00000135678 (CPM) | < 0.001 | 0.005 |
| ENSG00000261780 (LOC100505817) | < 0.001 | 0.006 |
| ENSG00000012223 (LTF) | < 0.001 | 0.006 |
| ENSG00000214711 (CAPN14) | < 0.001 | 0.006 |
| ENSG00000065618 (Unclassified) | < 0.001 | 0.006 |
| ENSG00000186212 (SOWAHB) | < 0.001 | 0.006 |
| ENSG00000163207 (IVL) | < 0.001 | 0.007 |
| ENSG00000134757 (DSG3) | < 0.001 | 0.007 |
| ENSG00000267283 (Unclassified) | < 0.001 | 0.007 |
| ENSG00000152292 (SH2D6) | < 0.001 | 0.008 |
| ENSG00000117507 (FMO6P) | < 0.001 | 0.009 |
| ENSG00000100146 (SOX10) | < 0.001 | 0.009 |
| ENSG00000184867 (ARMCX2) | < 0.001 | 0.009 |
| ENSG00000214049 (UCA1) | < 0.001 | 0.009 |
| ENSG00000131771 (PPP1R1B) | < 0.001 | 0.009 |
| ENSG00000149575 (SCN2B) | < 0.001 | 0.009 |
| ENSG00000163751 (CPA3) | < 0.001 | 0.010 |
| ENSG00000228639 (LOC102723505) | < 0.001 | 0.010 |
| ENSG00000274925 (Unclassified) | < 0.001 | 0.010 |
| ENSG00000184908 (CLCNKB) | < 0.001 | 0.011 |
| ENSG00000235437 (LINC01278) | < 0.001 | 0.011 |
| ENSG00000136155 (SCEL) | < 0.001 | 0.011 |
| ENSG00000142224 (IL19) | < 0.001 | 0.012 |
| ENSG00000149294 (NCAM1) | < 0.001 | 0.013 |
| ENSG00000280832 (Unclassified) | < 0.001 | 0.013 |
| ENSG00000189334 (S100A14) | < 0.001 | 0.013 |
| ENSG00000188803 (SHISA6) | < 0.001 | 0.014 |
| ENSG00000186038 (HTR3E) | < 0.001 | 0.015 |
| ENSG00000134339 (SAA2) | < 0.001 | 0.015 |
| ENSG00000156535 (CD109) | < 0.001 | 0.015 |
| ENSG00000146070 (PLA2G7) | < 0.001 | 0.015 |
| ENSG00000131650 (KREMEN2) | < 0.001 | 0.015 |
| ENSG00000113231 (PDE8B) | < 0.001 | 0.016 |
| ENSG00000255389 (Unclassified) | < 0.001 | 0.016 |
| ENSG00000254427 (LOC101928812) | < 0.001 | 0.016 |
| ENSG00000160183 (TMPRSS3) | < 0.001 | 0.017 |
| ENSG00000268230 (Unclassified) | < 0.001 | 0.017 |
| ENSG00000008277 (ADAM22) | < 0.001 | 0.018 |
| ENSG00000211935 (Unclassified) | < 0.001 | 0.018 |
| ENSG00000109684 (CLNK) | < 0.001 | 0.018 |
| ENSG00000124466 (LYPD3) | < 0.001 | 0.018 |
| ENSG00000133392 (MYH11) | < 0.001 | 0.018 |
| ENSG00000137440 (FGFBP1) | < 0.001 | 0.018 |
| ENSG00000188505 (NCCRP1) | < 0.001 | 0.018 |
| ENSG00000197838 (CYP2A13) | < 0.001 | 0.018 |
| ENSG00000242372 (EIF6) | < 0.001 | 0.018 |
| ENSG00000181524 (Unclassified) | < 0.001 | 0.019 |
| ENSG00000141682 (PMAIP1) | < 0.001 | 0.019 |
| ENSG00000167614 (TTYH1) | < 0.001 | 0.020 |
| ENSG00000198074 (AKR1B10) | < 0.001 | 0.021 |
| ENSG00000120907 (ADRA1A) | < 0.001 | 0.022 |
| ENSG00000173406 (DAB1) | < 0.001 | 0.022 |
| ENSG00000269821 (KCNQ1OT1) | < 0.001 | 0.022 |
| ENSG00000109193 (SULT1E1) | < 0.001 | 0.022 |
| ENSG00000168394 (TAP1) | < 0.001 | 0.022 |
| ENSG00000010438 (PRSS3) | < 0.001 | 0.022 |
| ENSG00000149654 (CDH22) | < 0.001 | 0.022 |
| ENSG00000171840 (NINJ2) | < 0.001 | 0.022 |
| ENSG00000132535 (DLG4) | < 0.001 | 0.022 |
| ENSG00000146904 (EPHA1) | < 0.001 | 0.022 |
| ENSG00000184194 (GPR173) | < 0.001 | 0.023 |
| ENSG00000006016 (CRLF1) | < 0.001 | 0.025 |
| ENSG00000213937 (CLDN9) | < 0.001 | 0.025 |
| ENSG00000169860 (P2RY1) | < 0.001 | 0.025 |
| ENSG00000135407 (AVIL) | < 0.001 | 0.025 |
| ENSG00000171101 (SIGLEC17P) | < 0.001 | 0.026 |
| ENSG00000086289 (EPDR1) | < 0.001 | 0.027 |
| ENSG00000151892 (GFRA1) | < 0.001 | 0.027 |
| ENSG00000163737 (PF4) | < 0.001 | 0.027 |
| ENSG00000172594 (SMPDL3A) | < 0.001 | 0.027 |
| ENSG00000146276 (GABRR1) | < 0.001 | 0.028 |
| ENSG00000257594 (GALNT4) | < 0.001 | 0.028 |
| ENSG00000145681 (HAPLN1) | < 0.001 | 0.028 |
| ENSG00000035720 (STAP1) | < 0.001 | 0.029 |
| ENSG00000089127 (OAS1) | < 0.001 | 0.029 |
| ENSG00000153294 (ADGRF4) | < 0.001 | 0.029 |
| ENSG00000236256 (DIAPH2-AS1) | < 0.001 | 0.029 |
| ENSG00000197191 (CYSRT1) | < 0.001 | 0.029 |
| ENSG00000168453 (HR) | < 0.001 | 0.029 |
| ENSG00000206075 (SERPINB5) | < 0.001 | 0.029 |
| ENSG00000223984 (Unclassified) | < 0.001 | 0.029 |
| ENSG00000269376 (Unclassified) | < 0.001 | 0.029 |
| ENSG00000131015 (ULBP2) | < 0.001 | 0.030 |
| ENSG00000166589 (CDH16) | < 0.001 | 0.030 |
| ENSG00000244675 (Unclassified) | < 0.001 | 0.030 |
| ENSG00000140287 (HDC) | < 0.001 | 0.031 |
| ENSG00000026559 (KCNG1) | < 0.001 | 0.031 |
| ENSG00000197641 (SERPINB13) | < 0.001 | 0.031 |
| ENSG00000201772 (SNORA5C) | < 0.001 | 0.031 |
| ENSG00000015479 (MATR3) | < 0.001 | 0.033 |
| ENSG00000115468 (EFHD1) | < 0.001 | 0.035 |
| ENSG00000270164 (LINC01480) | < 0.001 | 0.035 |
| ENSG00000271964 (Unclassified) | < 0.001 | 0.036 |
| ENSG00000126233 (SLURP1) | < 0.001 | 0.037 |
| ENSG00000272161 (Unclassified) | < 0.001 | 0.038 |
| ENSG00000159527 (PGLYRP3) | < 0.001 | 0.038 |
| ENSG00000162913 (C1orf145) | < 0.001 | 0.038 |
| ENSG00000186190 (BPIFB3) | < 0.001 | 0.038 |
| ENSG00000150281 (CTF1) | < 0.001 | 0.043 |
| ENSG00000196263 (ZNF471) | < 0.001 | 0.043 |
| ENSG00000221890 (NPTXR) | < 0.001 | 0.044 |
| ENSG00000259342 (Unclassified) | < 0.001 | 0.045 |
| ENSG00000183780 (SLC35F3) | < 0.001 | 0.047 |
| ENSG00000197415 (VEPH1) | < 0.001 | 0.048 |
| ENSG00000173432 (SAA1) | < 0.001 | 0.049 |
| * *P*-value for protein data represents ANOVA test *p*-value (across all three time points). Transcriptome *p*-value represents unadjusted *p*-value from DESeq2 analysis (time i vs. ii).  ** Proteome *p*-adjusted represents post-hoc pairwise testing of time i vs. ii using Tukey's test. Transcriptome *p*-adjusted represents FDR adjusted *p*-values from DESeq2 analysis (time i vs. ii). | | |
