# Supplementary material for: Multiomic analysis identifies natural intrapatient temporal variability and changes in response to systemic corticosteroid therapy in chronic rhinosinusitis
Source: Immun Inflamm Dis. 2020 Nov 21;9(1):90–107. doi: 10.1002/iid3.349 (PMC7860613; doi:10.1002/iid3.349)
Supplement: Supplementary file 3 — Supporting information. [file IID3-9-90-s003.docx]

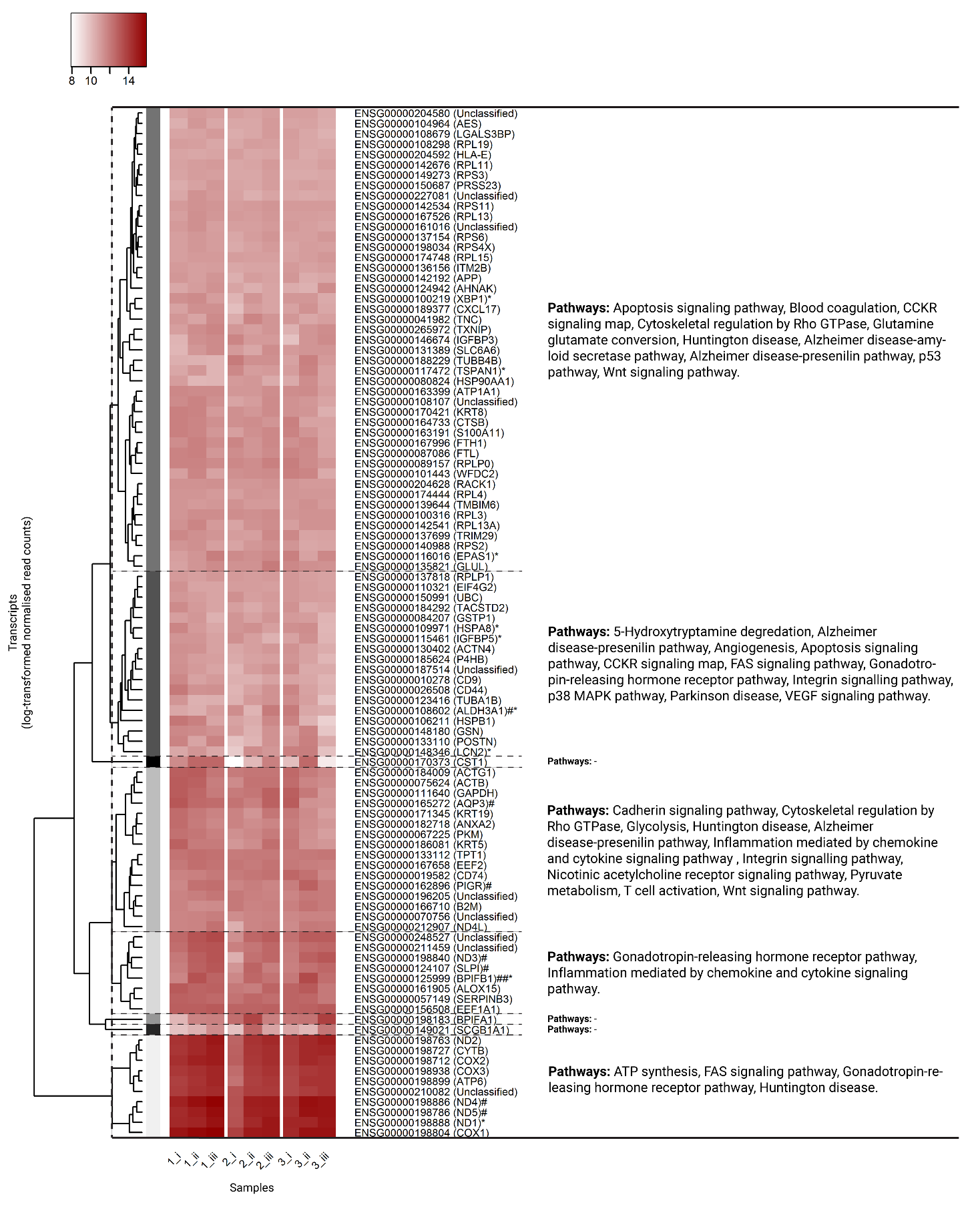


**Supporting Information 3: Figure S1.** Most abundant transcripts and associated PANTHER pathways.

Heat map of log-transformed normalised read counts for the 100 most abundant transcripts. Transcripts are ordered via hierarchical clustering based on Bray-Curtis dissimilarity. Transcripts are divided into 8 clusters (represented by the grey-scale colour coding at branch tips), and associated pathways for each cluster are presented on the right. Samples represent three patients (1, 2, and 3) at three time points over two consecutive weeks (i, ii, and iii). # = unadjusted *p*-value < 0.05 and ## = FDR adjusted *p*-value < 0.05 in testing between time points i and ii (natural variability). * = unadjusted *p*-value < 0.05 and ** = FDR adjusted *p*-value < 0.05 in testing between time points ii and iii (response to corticosteroids).


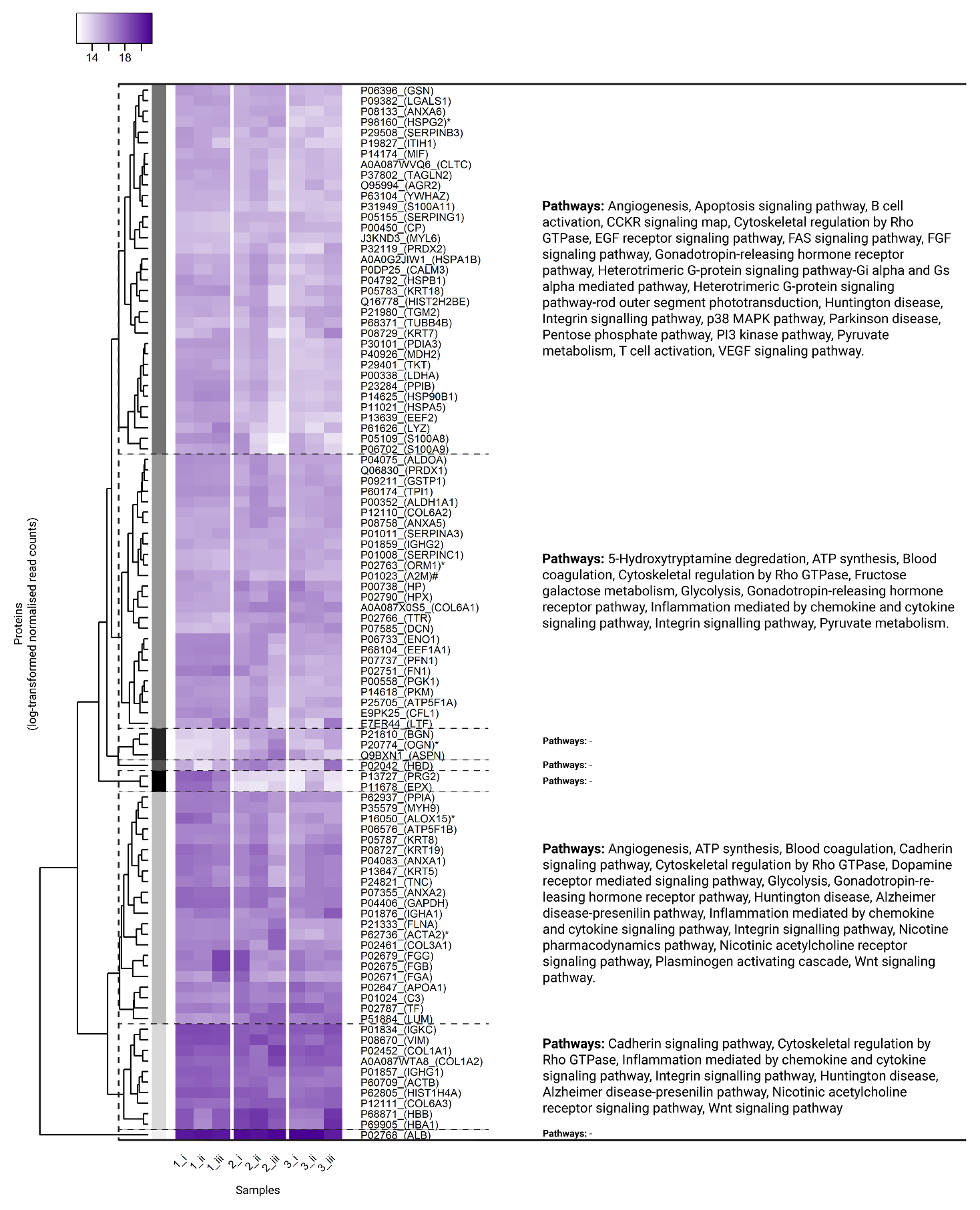


**Supporting Information 3: Figure S2.** Most abundant proteins and associated PANTHER pathways.

Heat map of log-transformed normalised read counts for the 100 most abundant proteins. Proteins are ordered via hierarchical clustering based on Bray-Curtis dissimilarity. Proteins are divided into 8 clusters (represented by the grey-scale colour coding at branch tips), and associated pathways for each cluster are presented on the right. Samples represent three patients (1, 2, and 3) at three time points over two consecutive weeks (i, ii, and iii). # = unadjusted *p*-value < 0.05 and ## = FDR adjusted *p*-value < 0.05 in testing between time points i and ii (natural variability). * = unadjusted *p*-value < 0.05 and ** = FDR adjusted *p*-value < 0.05 in testing between time points ii and iii (response to corticosteroids).


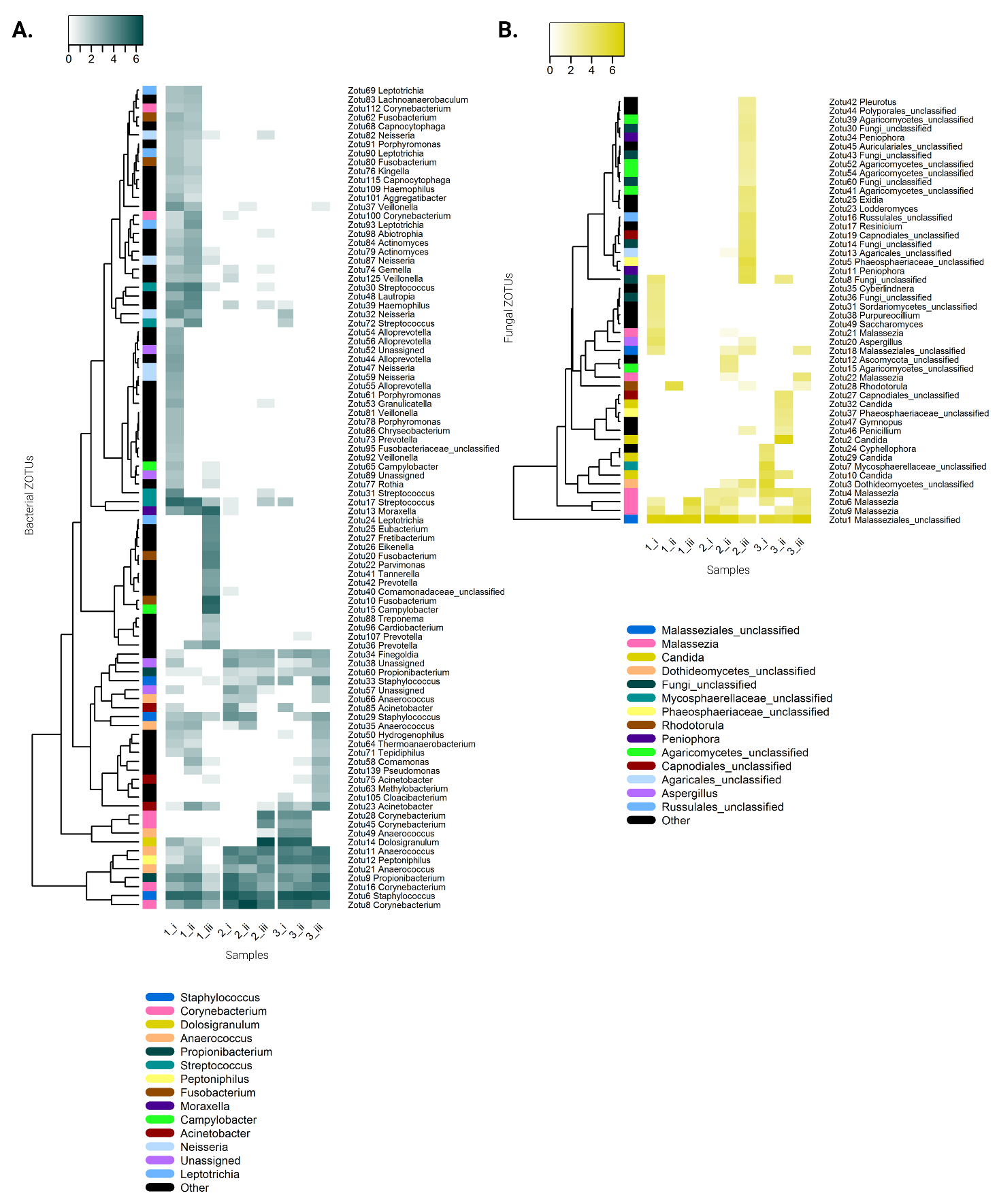


**Supporting Information 3: Figure S3.** Polyp-associated bacterial and fungal communities.

**A.** Log-transformed relative abundances of bacterial ZOTUs based on 16S rRNA amplicon sequencing. **B.** Log-transformed relative abundances of fungal ZOTUs based on ITS2 marker amplicon sequencing. Samples represent three patients (1, 2, and 3) sampled at three time points over two consecutive weeks (i, ii, and iii). ZOTUs are ordered based on hierarchical clustering. ZOTUs from the 14 most abundant genera (or higher rank, if unclassified to genus) are colour-coded next to the branch tips, with all other genera coloured black.


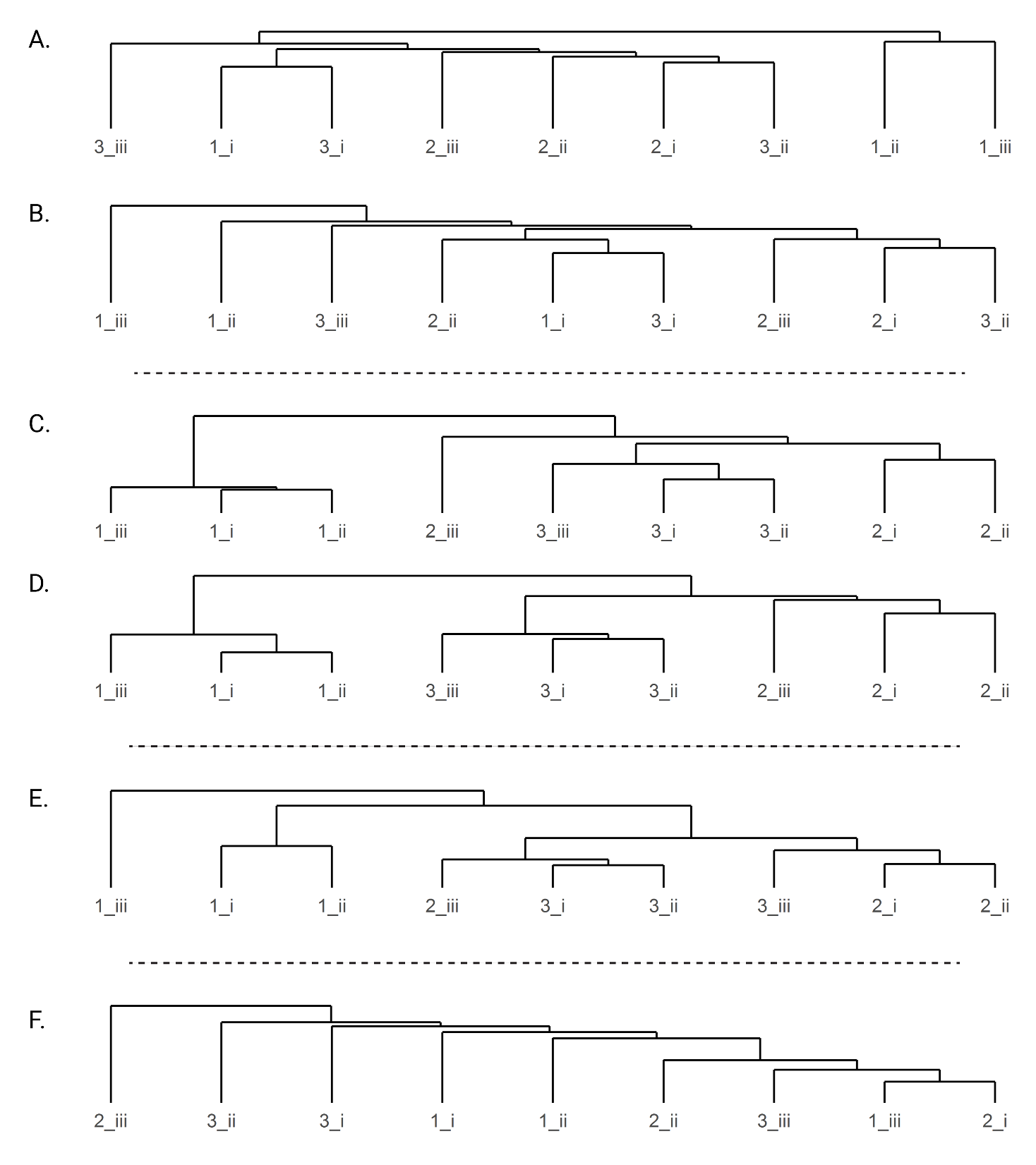


**Supporting Information 3: Figure S4.** Hierarchical clustering analyses.

UPGMA hierarchical clustering of samples based on Bray-Curtis dissimilarity. Samples are three patients (1, 2, and 3), with sampling at three time points over two consecutive weeks (time points i, ii, and iii). **A.** Transcriptome, full data set. **B.** Transcriptome, pathways_subset data. **C.** Proteome, full data set. **D.** Proteome, pathways_subset data. **E.** Bacterial community (16S rRNA amplicon sequencing) data. **F.** Fungal community (ITS2 amplicon sequencing) data.


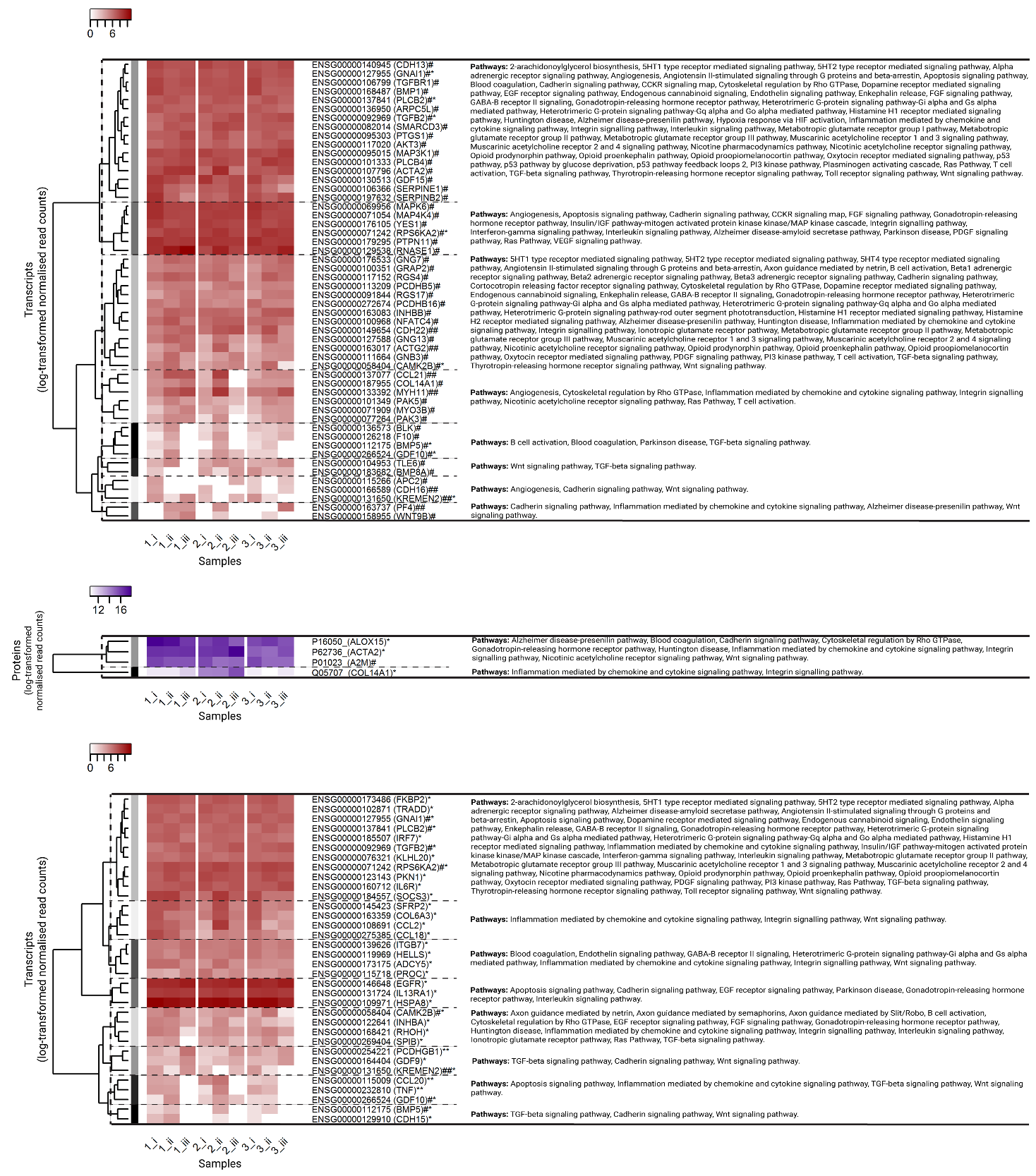


**A.**

**C.**

**B.**

**Supporting Information 3: Figure S5.** Subset_pathways (PANTHER pathways of interest) transcripts and proteins that differed in testing between times i and ii (natural variability) and ii and iii (response to corticosteroids).

Heat maps of log-transformed normalised read counts for: **A.** transcripts (DEG) that differed between times i and ii; **B.** proteins that differed between i and ii, or ii and iii; and **C.** transcripts (DEG) that differed between times ii and iii. Variables are ordered via hierarchical clustering based on Bray-Curtis dissimilarity. Variables are divided into clusters (represented by the grey-scale colour coding at branch tips), and associated pathways for each cluster are presented on the right. Samples represent three patients (1, 2, and 3) at three time points over two consecutive weeks (i, ii, and iii). # = unadjusted *p*-value < 0.05 and ## = FDR adjusted *p*-value < 0.05 in testing between time points i and ii (natural variability). * = unadjusted *p*-value < 0.05 and ** = FDR adjusted *p*-value < 0.05 in testing between time points ii and iii (response to corticosteroids).


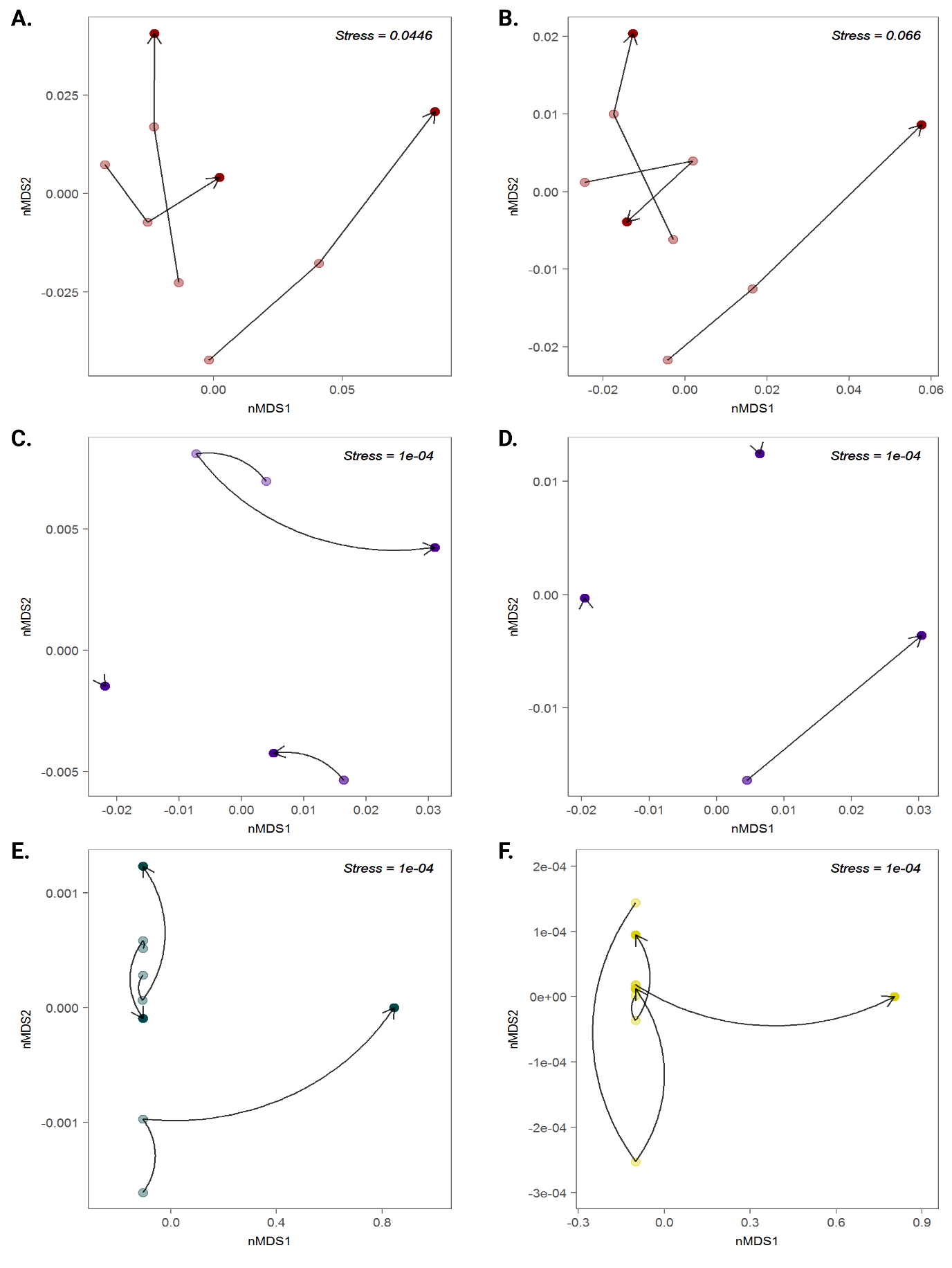


**Supporting Information 3: Figure S6.** Ordination analyses.

Non-metric multidimensional scaling plots based on Bray-Curtis dissimilarity for: **A.** transcriptome, full data set; **B.** transcriptome, pathways_subset data; **C.** proteome, full data set; **D.** proteome, pathways_subset data; **E.** bacterial community data; and **F.** fungal community data. Vector lines join the three samples from each individual patient, with arrows indicating the time course. Curved lines are for visualisation purposes, but do not represent a curved trajectory of change over the week between each time point. The final arrow point (darker coloured points) indicates the post-corticosteroid therapy time point.
